# A pair of primary colorectal cancer-derived and corresponding synchronous liver metastasis-derived organoid cell lines

**DOI:** 10.18632/aging.205595

**Published:** 2024-02-24

**Authors:** Fangling Cheng, Pengcheng Li, Sanpeng Xu, Chao Zhang, Huifang Liang, Zeyang Ding

**Affiliations:** 1 Hepatic Surgery Centre, Tongji Hospital, Tongji Medical College, Huazhong University of Science and Technology, Wuhan 430030, China; 2 Hubei Key Laboratory of Hepato-Pancreato-Biliary Diseases, Wuhan 430030, China; 3 Institute of Pathology, Tongji Hospital, Tongji Medical College, Huazhong University of Science and Technology, Wuhan 430030, China

**Keywords:** 3D organoids, liver metastasis model, IC_50_ test, carcinoembryonic antigen

## Abstract

Proper preclinical models for the research of colorectal cancer (CRC) and CRC liver metastases (CLM) are a clear and unmet need. Patient-derived organoids have recently emerged as a robust preclinical model, but are not available to all scientific researchers. Here, we present paired 3D organoid cell lines of CWH22 (CRC-derived) and CLM22 (CLM-derived) with sound background information and the short tandem repeats are identical to those of the normal tissue. Morphological and immunohistochemical staining, along with whole-exome sequencing (WES), confirmed that the organoids exhibited the same differentiation, molecular expression, and mutation status as the corresponding tumor tissue. Both organoids possessed mutated *APC*/*KRAS*/*SMAD4/CDKN1B/KMT2C* genes and wild-type *TP53* and *PIK3CA*; stably secreted the tumor markers CEA and CA19-9, and possessed sound proliferation rates *in vitro*, as well as subcutaneous tumorigenicity and liver metastatic abilities *in vivo*. IC_50_ assays confirmed that both cell lines were sensitive to 5-fluorouracil, oxaliplatin, SN-38, and sotorasib. WES and karyotype analyses revealed the genomic instability status as chromosome instability. The corresponding adherent cultured CWH22-2D/CLM22-2D cells were established and compared with commonly used CRC cell lines from the ATCC. Both organoids are publicly available to all researchers and will be useful tools for specific human CRC/CLM studies both *in vitro* and *in vivo.*

## INTRODUCTION

Colorectal cancer (CRC) is one of the most common malignant tumors in humans, accounting for ~10% of all cancer incidences and 9.4% of all cancer-related mortalities worldwide [1, 2]. The stage at diagnosis is the most important survival predictor; the 5-year relative survival rate ranges from 90%, for patients diagnosed with localized disease (stage I), to 14% for those with late-stage disease (stage IV) [3]. The liver is the most common metastatic organ in CRC. Approximately 20–25% of patients with CRC have liver metastases at the time of initial diagnosis, whereas 50% of postoperative CRC patients develop liver metastases [3–5]. Although radical surgical resection is an effective treatment for CRC liver metastases (CLM), less than 20% of patients are eligible for resection of such metastases, and all patients inevitably experience CLM recurrence [6]. Currently, systemic treatment for metastatic CRC relies primarily on the combination of chemotherapy with 5-fluorouracil (5-Fu), oxaliplatin, or irinotecan, in combination with targeted agents, such as cetuximab (for left-sided CRCs with wild-type *KRAS*/*NRAS*) and bevacizumab [7]. However, the overall survival of patients with CLM has not improved much in recent years. One major hurdle in the development of novel treatment regimens for CRC/CLM is the challenge of translating scientific findings from bench to bedside, mainly because most research models poorly replicate the heterogeneous behavior of CRC/CLM and, consequently, many drugs that perform well in CRC models fail in clinical trials.

Cell lines are attractive models for the study of malignant diseases. Currently, CRC/CLM cultures, especially publicly available cell lines, such as 2D cultured cell lines distributed by the American Type Culture Collection (ATCC; https://www.atcc.org), are useful tools for studying CRC formation and metastasis, developing new anti-tumor strategies, and identifying molecular markers in response to drugs. Although these cell lines have been widely used for decades and are publicly recognized, they have several drawbacks. For instance, *in vitro* subculture imposes selection pressure on cell lines, which can result in genetic drift over time [8]. Moreover, long-term cultures present a risk of cross-contamination with other cell lines [8, 9]. Thus, short tandem repeat (STR) authentication of already available cell lines and experiments with primary cell cultures are required to ensure the reliability of experimental results.

Since clonal heterogeneity is a characteristic of most human cancers [10], research models that fully reproduce the heterogeneity of primary tumors will be useful research tools. Patient-derived tumor organoids are cultures of tumor cells that capture the heterogeneity of morphological and genetic features of the original tumor and can be derived from individual patients with a high success rate and unlimited expansion potential [11–13]. Accumulating evidence has demonstrated that CRC/CLM-derived organoids can be used not only for exploring tumor biological characteristics, but also as preclinical models for predicting treatment responses [12, 14–17]. Organoids derived from paired CRC/CLM lesions have been reported in several articles [15, 18, 19]; however, their biological and molecular characteristics have not been fully explored for each patient, and they have not been widely used by all researchers.

Throughout this study, we presented paired CRC/CLM-derived organoids, named CWH22 (CRC-derived) and CLM22 (CLM-derived) organoid cell lines, which are suitable for common 3D organoid cultures with minimal additions to advanced Dulbecco’s modified Eagle medium (DMEM)/F12. Both organoid lines were subcultured more than 50 times and cryopreserved at different early passage stages. We also presented their biological characteristics, including proliferation rate, karyotype analysis, IC_50_ values for different drugs, and whole-exome sequencing (WES) data, as well as the pathological and histological characteristics of the original tumor and the subcutaneous/liver metastasis xenografts of these two cell lines in nude mice. Moreover, 2D adherent cultures of CWH22 (CWH22-2D) and CLM22 (CLM22-2D) cells were also established and cultured in high-glucose DMEM supplemented with 10% fetal bovine serum. The advantages of CWH22 and CLM22 organoid cell lines, as well as CWH22-2D and CLM22-2D cell lines, compared to traditional adherent cell lines from the ATCC were also demonstrated.

Since the earliest passages of CWH22 and CLM22 organoid cell lines, as well as CWH22-2D and CLM22-2D cell lines, were preserved at the China Center for Type Culture Collection, Wuhan, China (CCTCC), and are available to all researchers, they will contribute to the diversity of CRC cell lines, the exploration of the molecular mechanisms, and facilitate the screening and evaluation of anti-tumor drugs in CRC/CLM studies, rendering preclinical research more reliable.

## RESULTS

### Establishment of CWH22/CLM22 3D organoids

**Figure 1:**
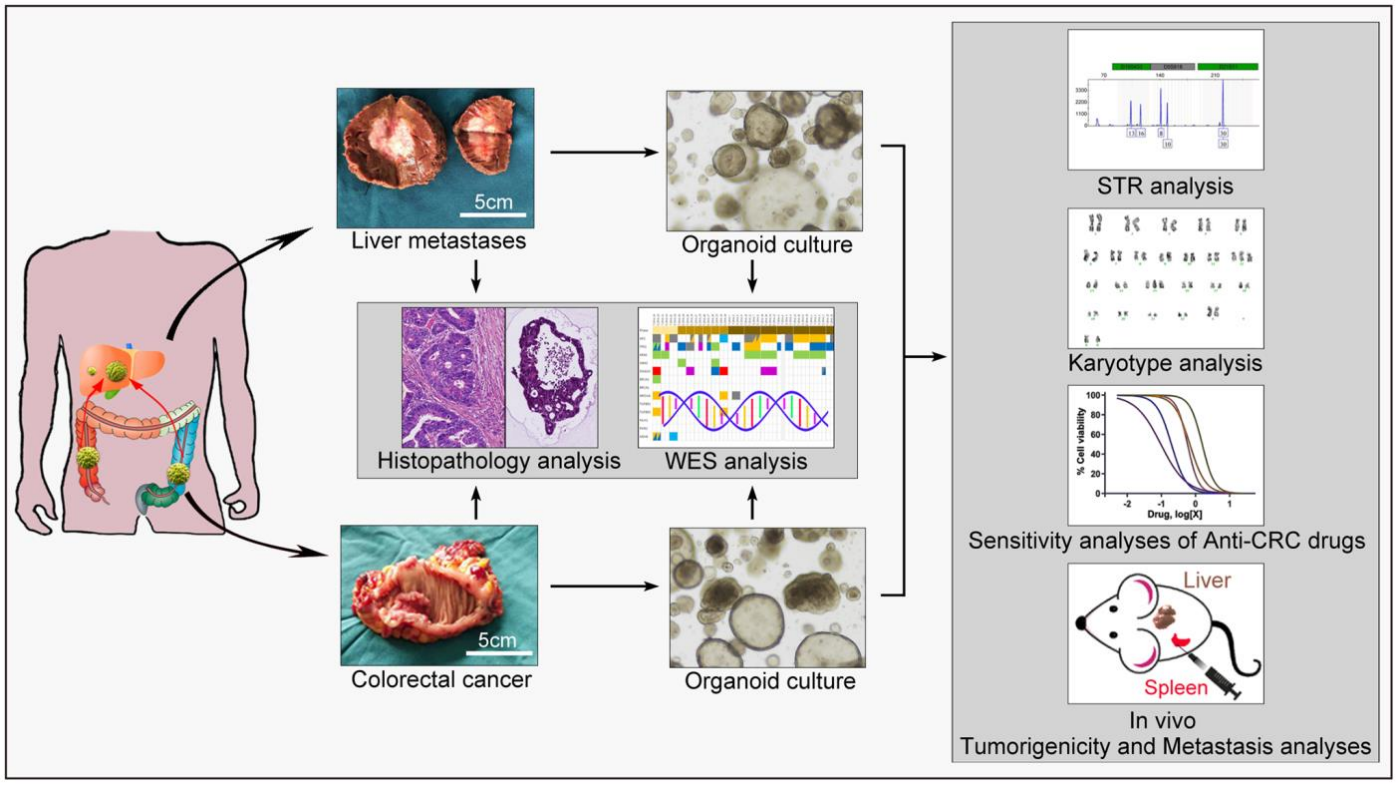
**Overview of the procedure.** Tissues from primary colorectal cancer and liver metastases were obtained and then divided into three parts: two parts were placed in liquid nitrogen and 4% paraformaldehyde, separately, for molecular and histopathological examinations, and the other part was immediately placed on ice and transported to the laboratory for tumor cell isolation and organoid culture. Whole-exome sequencing and histopathological analysis were conducted to determine the concordance between the tumor organoids and corresponding tumors. The organoids were subjected to STR, karyotype analysis, *in vitro* drug sensitivity tests, and *in vivo* animal model construction.

The experimental design of the procedure is summarized in Figure 1. The occupied lesions in the left liver lobe and ascending colon are presented in Supplementary Figure 1A, 1B, respectively. H&E and IHC staining indicated moderately differentiated adenocarcinomas with tubular structures of colorectal epithelial origin of colorectal epithelial origin (cytokeratin 20 (CK20)- and CDX2-positive) with wild-type p53, high proliferation rates (Ki67), and microsatellite stability (MSS; normal expression of MLH1, PMS2, MSH2, and MSH6 proteins; Supplementary Figure 1C). The treatment strategies and the response records are presented in Supplementary Figure 1D, 1F.

**Figure 2:**
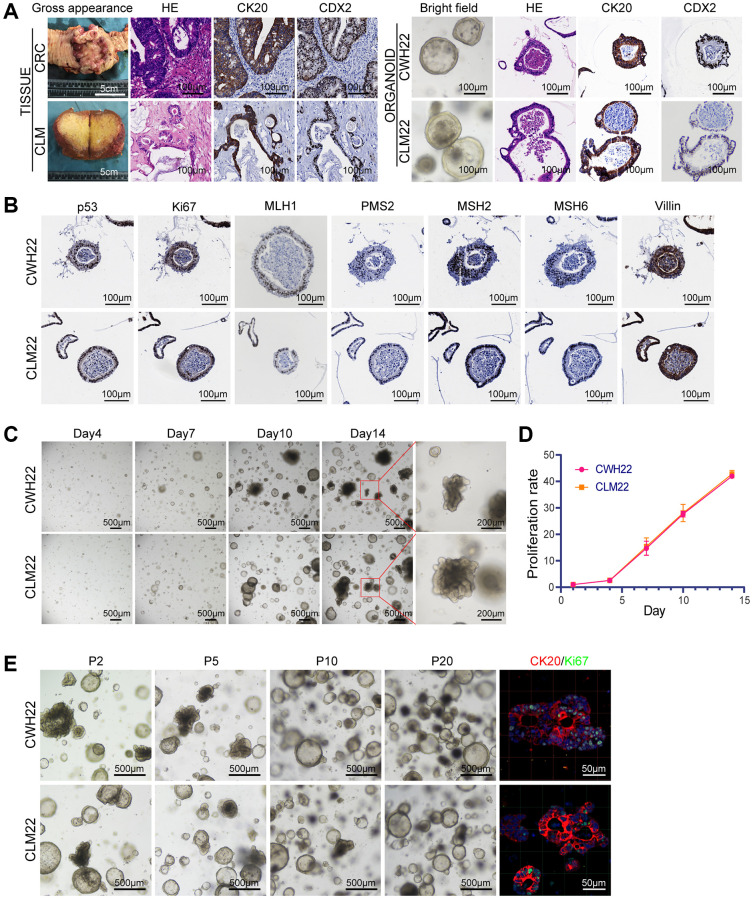
**CWH22 and CLM22 organoids maintain histological and proliferative characteristics of the corresponding tumors.** (**A**) H&E staining of the tumor organoids showing the resemblance of the organoids to the lumens and tubular structures of the primary tumor. IHC staining demonstrated consistent positive expression of CK20 and CDX2 in the organoids and corresponding tumors (scale bars, 100 μm). (**B**) IHC staining of CWH22 and CLM22 organoids demonstrating the consistent expression of p53, Ki67, MLH1, PMS2, MSH2, MSH6, and Villin, with the expression in CRC and CLM tissues (Supplementary Figure 1C). Scale bars, 100 μm. (**C**) Representative time course of CWH22 and CLM22 organoid growth for 14 days (scale bars, 500 μm) and local field amplification (scale bars, 200 μm). (**D**) Analysis of the proliferation rates of CWH22 and CLM22 organoids over time using 3D cell viability tests. (**E**) Bright-field images showing the morphology of CWH22 and CLM22 organoids at the 2nd, 5th, 10th, and 20th passages (P2, P5, P10, and P20, respectively; scale bars, 500 μm), and the expression of CK20 and Ki67 in representative differentiated organoids from CWH22 and CLM22 (scale bars, 50 μm).

During culture, both CRC- and CLM-derived organoids, namely CWH22 and CLM22, proliferated and differentiated well. The gross appearance of the CRC and CLM tissues, as well as bright-field images of the organoids, are presented in Figure 2A. H&E staining and IHC staining revealed morphological (e.g., thin- and thick-walled cystic structures) and protein expression similarities between CWH22/CLM22 organoids and the corresponding tumor tissues (Figure 2A, 2B; Supplementary Figure 1C). Both CWH22 and CLM22 organoids circumvented replicative senescence, acquired the ability to sustain unlimited proliferation in culture (passaged more than 50 times), and were cryopreserved at different early passage stages. Two movie recordings (Supplementary Videos 1 and 2) show the bright-field morphologies of CWH22 and CLM22 ten days after recovery, with continuous zoom at different focal surfaces, demonstrating that these organoids can withstand freezing, storage, and thawing.

### Proliferation, morphological stability, and identification of CWH22/CLM22 organoid cells

Proliferation and tumorigenicity are basic features of primary and organoid cell lines, making them popular models in cancer research. CWH22 and CLM22 organoids grow rather slowly during the first five days, owing to the intentionally low number of cells initially seeded into each well; this was done to provide more 3D space in the BME for organoid growth and differentiation (Figure 2C). Subsequently, the organoids grew rapidly, and the total cell viability on the 14th day was 40 times greater than that on the first day. Moreover, the proliferation rates of this paired organoids (CWH22 and CLM22) did not differ (Figure 2D).

Apart from the proliferation conditions in the 3D culture system, stable organoid morphology was also maintained over long periods. Luminal and glandular epithelioid folds were evident at different passages (P2, P5, P10, and P20) in both CWH22 and CLM22 organoids, which maintained the structural heterogeneity well (Figure 2E). Immunofluorescence staining of the differentiated structures in CWH22 and CLM22 organoids was performed in the 20th generation, revealing that both organoids were positive for CK20, which is normally expressed in luminal cells of the colonic mucosa, and Ki67, a proliferation marker (Figure 2E).

Since CWH22 and CLM22 meet the conditions of being cell lines and possess good tumorigenicity (see the following sections), the 4th generation of these organoid cell lines were deposited at the CCTCC (No. C202218 and C202219) after testing negative for bacterial and mycoplasma contamination. The STR authentications of 21 locations (including the nine ATCC-required loci) from these two organoid cell lines and the corresponding normal mucosa tissue were assessed by the CCTCC, and a complete match was observed (Table 1). Ample evidence shows that cross-contamination or phenotypic drift of cells in culture can generate irreproducible or misleading data, and many scientific journals require cell identification based on DNA analysis of samples and cell lines used [20, 21]. The STR profile of this case differed from that of all cell lines available in different cell banks (http://cellresource.cn/str/default.aspx).

**Table 1:** Information regarding 21 short-tandem repeat (STR) loci of the organoids and corresponding patient tissue.

**STR loci**	**Normal tissue**	**CWH22**	**CLM22**
D19S433	13,16	13,16	13,16
D5S818	8,10	8,10	8,10
D21S11	30,30	30,30	30,30
D18S51	13,15	13,15	13,15
D6S1043	13,18	13,18	13,18
AMEL	X, X	X, X	X, X
D3S1358	15,16	15,16	15,16
D13S317	8,12	8,12	8,12
D7S820	8,10	8,10	8,10
D16S539	9,12	9,12	9,12
CSF1PO	11,12	11,12	11,12
Penta D	10,13	10,13	10,13
D2S441	11,11	11,11	11,11
vWA	17,17	17,17	17,17
D8S1179	13,13	13,13	13,13
TPOX	8,11	8,11	8,11
Penta E	16,17	16,17	16,17
TH01	9,9	9,9	9,9
D12S391	20,24	20,24	20,24
D2S1338	23,27	23,27	23,27
FGA	19,23	19,23	19,23

### WES of CWH22/CLM22 organoids and corresponding tissues

**Figure 3:**
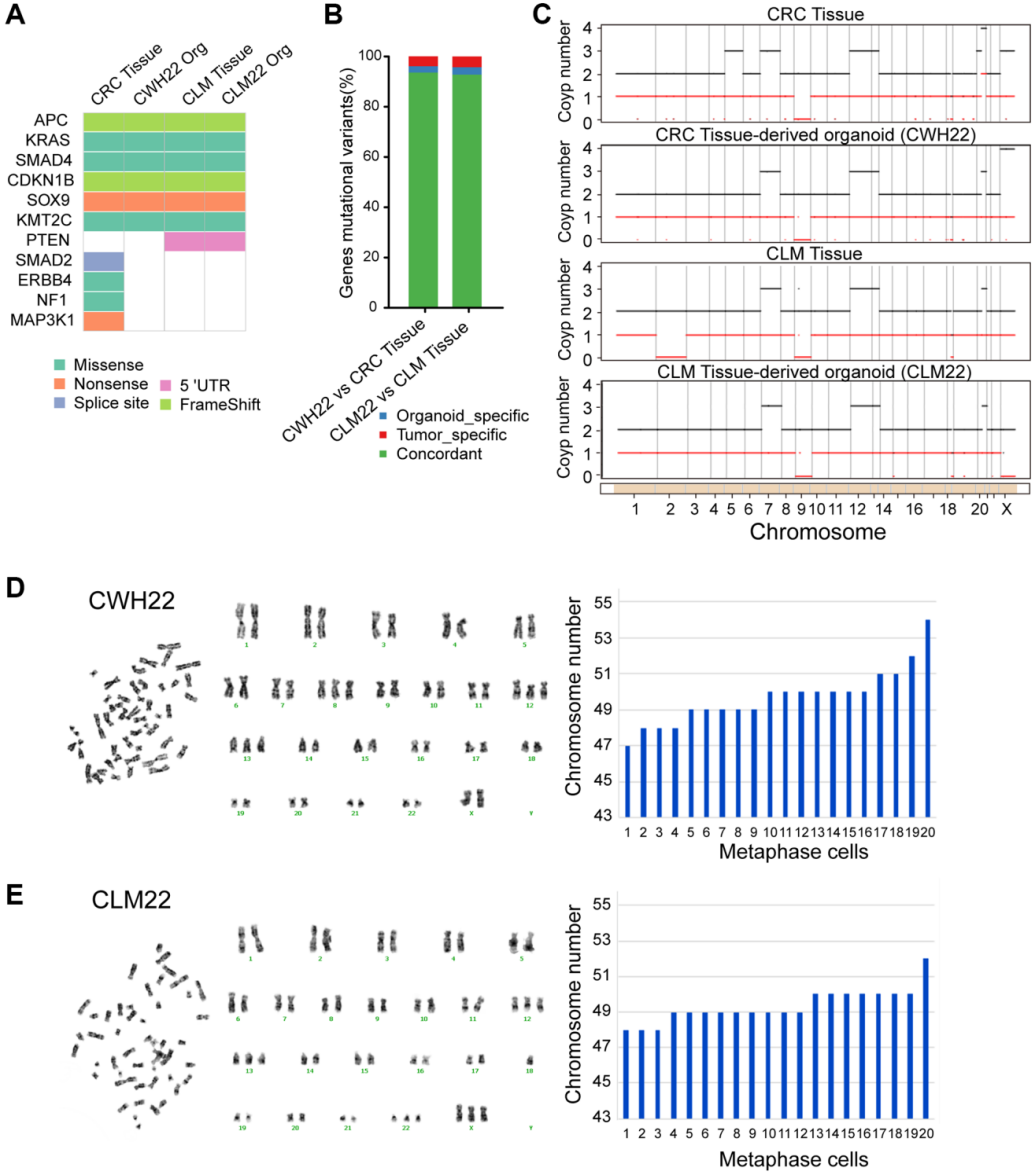
**Whole-exome sequencing (WES) and chromosome karyotype analysis of CWH22 and CLM22 organoids.** (**A**) Overview of somatic mutations found in CRC/CLM tissues and CWH22/CLM22 organoids. (**B**) Histogram showing the concordance (percentage) of SNVs between CWH22/CLM22 organoids and corresponding CRC/CLM tissues. (**C**) The ordinate represents the multiple copy variations, the black line represents the multiple total copy number variations, and the red line represents the multiple minor copy number variations. When the value of the black line is >2, the copy number increases; when it is <2, the copy number decreases. (**D**, **E**) Chromosome karyotype pairing from one representative karyotype analysis and the chromosome numbers of 20 different metaphase cells of (**D**) CWH22 and (**E**) CLM22.

Organoids derived from cancer patients recapitulate the genomic profiles of the corresponding tumors, including DNA copy number variations and mutations [12, 22, 23]. Thus, genomic DNA was isolated, and WES was performed on the two organoid cell lines, the corresponding normal mucosa, CRC, and CLM tissues (Figure 3A–3C). The organoids retained the somatic mutational spectrum observed in the patient’s tumors, and these were among the top mutated genes of CRC with liver metastases [24] (https://www.cbioportal.org/study/summary?id=crc_msk_2017; Figure 3A). Like those in the original tumors, CWH22 and CLM22 organoids harbored mutations in pathways important for CRC initiation and progression, including the WNT (*APC* mutation), RAS-MAPK (*KRAS* mutation), and TGF-β (*SMAD4* mutation) pathways, as well as *KMT2C* and *CDKN1B* mutations (Figure 3A). Thus, the CWH22 and CLM22 organoid cell lines are representative of *APC*, *KRAS*, *SMAD4*, *CDKN1B*, and *KMT2C* quintuple-mutated CRC cell lines with wild-type *TP53* and *PIK3CA* (Table 2). The organoids retained most somatic variants present in the corresponding original tissue (CWH22 vs. CRC tissue: 93.56%; CLM22 vs. CLM tissue: 92.74%; Figure 3B). Furthermore, the original copy number alterations at the genome-wide level were recapitulated in CWH22 and CLM22 organoids (Figure 3C). These data demonstrated that the CWH22 and CLM22 organoid lines are highly consistent with the corresponding tumor tissues at the genomic level.

**Table 2:** List of certain specific mutant genes based on whole-exome sequencing (WES) of organoid cells.

**Gene**	**Location**	**Mutation type**	**Transcript**	**Mutation Frequency**	
				**CWH22**	**CLM22**
*APC*	5q22.2	Frame Shift	NM_000038.5:p.Tyr935fs/c.2805_2806delCA	0.427	0.527
*APC*	5q22.2	Frame Shift	NM_000038.5:p.Phe1491fs/c.4473delT	0.382	0.566
*KRAS*	12p12.1	Missense	NM_033360.3:p.Gly12Cys/c.34G>T	0.607	0.662
*SMAD4*	18q21.2	Missense	p.Gln3158His/c.9474G>T	0.987	0.985
*CDKN1B*	12p13.1	Frame Shift	NM_004064.4:p.Ser110fs/c.323_329dupATGTCAG	0.666	0.66
*SOX9*	17q24.3	Nonsense	NM_000346.3:p.Glu28^*^/c.82G>T	0.507	0.512
*KMT2C*	7q36.1	Missense	NM_170606.2:p.Gln3158His/c.9474G>T	0.374	0.313

^*^Refers to the last changed amino acid.

### Karyotyping of CWH22/CLM22 organoids

According to WES results and analysis system of BGI, the tumor mutation burden (TMB, Mut/Mb)/microsatellite instability (MSI) sensor results for CWH22, CRC, CLM22, and CLM samples were 119.85/0.57, 106.85/0.1, 146.53/0.52, and 166.78/0.16, respectively, indicating the absence of MSI in all samples (MSI score threshold, 3.5). Combined with the normal expression levels of MLH1, PMS2, MSH2, and MSH6 proteins in tumor tissues and organoids (Supplementary Figure 1C and Figure 2B), MSS was confirmed in CWH22 and CLM22 organoids. Since MSI and chromosomal instability (CIN) are frequent events in cancer that result in the accumulation of oncogenes and inactivation of tumor suppressor genes [25–28], facilitating tumorigenesis and progression, organoid cells from the fifth passage were separately harvested for G-banding karyotype analysis. The chromosomal numbers ranged from 47 to 54 in CWH22 organoid cells and from 48 to 52 in CLM22 organoid cells while counting 20 karyotypes (Figure 3D, 3E). Chromosome karyotype pairing revealed that extra copies of different chromosomes were rather common, especially chromosomes 12 and 13 (Figure 3D, 3E), which are also stably presented in the copy number variation (CNV) at the genome-wide level (Figure 3C). Thus, from these data, we conclude that CIN was the main cause of genomic instability in these two organoids.

*KRAS* mutation, a common phenomenon in cancer, is found in ~40% of CRCs [29]. As this gene is located on the short arm of chromosome 12 (12p12.1, Table 2), both CNV (Figure 3C), and karyotype analyses of CWH22 and CLM22 (Figure 3D, 3E) suggested that three copies of chromosome 12 are common in these two organoids. Therefore, each organoid most likely has three copies of the KRAS gene. Moreover, the percentages of these two organoids with *KRAS* G12C mutations, as indicated by WES, were 60.7% and 66.2% respectively (Table 2); we speculate that *KRAS* G12C mutation is heterozygous and most likely to be mutation-positive on two chromosomes and wild-type on the third chromosome.

### Drug response of CWH22 and CLM22 organoids

Since metastasis is a key contributor to CRC-associated deaths and effective therapies are lacking, the exploration of treatment strategies is of great importance to improve patient outcomes [30]. The use of genomics to guide cancer therapies was promoted after the widespread use of genetic testing. However, this approach has been less successful than anticipated, and less than 7% of patients in 2018 were estimated to benefit from such precision oncology [31]. Organoids have been demonstrated to be effective tools for predicting CRC responses to different treatments in clinical practice [12, 15, 17, 18, 32]; thus, we selected several compounds to determine the sensitivity of CWH22 and CLM22 organoids to these drugs.

**Figure 4:**
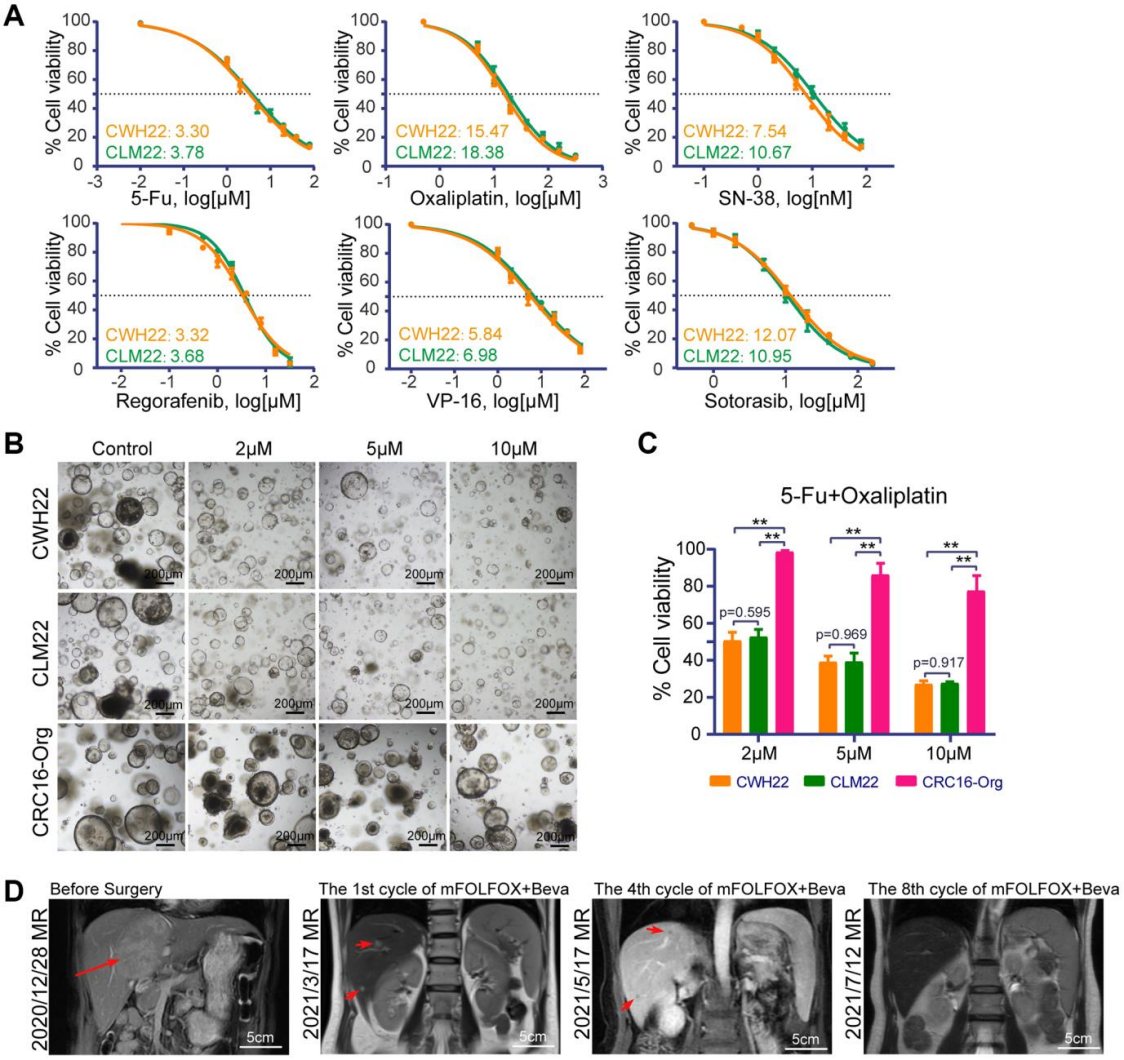
**Drug treatment sensitivities of CWH22 and CLM22 organoids to traditional chemotherapies.** (**A**) Dose-response curves of CWH22 and CLM22 organoids exposed to increasing concentrations of 5-fluorouracil, oxaliplatin, SN-38, regorafenib, VP-16, and sotorasib, and the half maximal inhibitory concentrations (IC_50_) of each drug. (**B**) Representative images of CWH22 and CLM22 organoids exposed to three concentrations of 5-Fu and oxaliplatin (2 μM, 5 μM, and 10 μM; scale bars, 200 μm). (**C**) Quantitative analysis of CWH22, CLM22, and CRC16-Org cell viability following exposure to different concentrations of FO combined treatment (2 μM, 5 μM, and 10 μM; *n* = 3; data represented as the mean ± SD, ^**^p < 0.01). (**D**) Preoperative and postoperative MRI coronal images of liver lesions during the first eight cycles treatment with mFOLFOX + bevacizumab strategies (scale bars, 5 cm).

5-Fu, oxaliplatin, SN-38, regorafenib, etoposide (VP-16), and sotorasib are common drugs used for CRC treatment and preclinical study. The dose-response curves and IC_50_ values of CWH22 and CLM22 organoids exposed to the selected drugs, as shown in Figure 4A, were consistent with previous reports indicating no significant difference in the drug sensitivity of CRC and paired LM organoids from the same patient for certain drugs [15, 18]. The IC_50_ values of 5-Fu, oxaliplatin and SN-38 for CWH22/CLM22 organoids were 3.3/3.78 μM, 15.47/18.38 μM and 7.54/10.67 nM respectively, indicating that CWH22/CLM22 organoids were more sensitive to these drugs than LoVo, SW620, T84, and SW480 cells, according to the data from the Genomics of Drug Sensitivity in Cancer database (GDSC; https://www.cancerrxgene.org/; 10–415 μM for 5-Fu; 28–35 μM for oxaliplatin; 0.0139–39 μM for SN-38). Next, we conducted combined treatment with 5-Fu + oxaliplatin (FO, 1:1) according to the patient’s treatment regimen. Pictures of CWH22, CLM22, and another resistant organoid CRC16-Org (pre-experimental results indicating original resistance to 5-Fu and oxaliplatin) exposed to three concentrations of FO (2 μM, 5 μM, and 10 μM) are shown in Figure 4B. Further analysis revealed higher sensitivity of CWH22 and CLM22 organoids to FO treatment compared to CRC16-Org (Figure 4C; the organoid cell viability decreased by 60.96 ± 3.26%, 60.75 ± 4.74%, and 13.58 ± 5.99%, respectively, upon treatment with 5 μM FO). Clinically, the shrinkage of liver metastatic lesions demonstrated apparent sensitivity to the first eight cycles of the mFOLFOX + bevacizumab treatment strategy; the coronal image records of the liver during the treatment are presented in Figure 4D.

**Figure 5:**
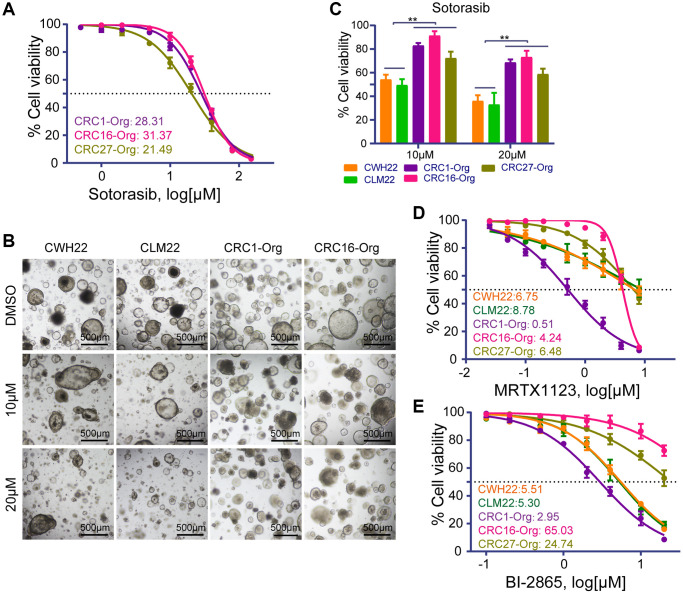
**Drug treatment sensitivities of different organoids to KRAS G12C, KRAS G12D and pan-KRAS inhibitors.** (**A**) Dose-response curves of KRAS G12D-mutated organoids (CRC1-Org and CRC16-Org) and RAS-unmutated organoid (CRC27-Org) exposed to increasing concentrations of sotorasib (a KRAS G12C inhibitor), and the corresponding IC_50_ values. (**B**) Representative images of CWH22, CLM22, CRC1-Org, and CRC16-Org organoids exposed to sotorasib at concentrations of 10 μM and 20 μM (scale bars, 200 μm). (**C**) Quantitative analysis of the viability of CWH22, CLM22, CRC1-Org, CRC16-Org and CRC27-Org (with different KRAS mutation backgrounds) following exposure to 10 μM and 20 μM sotorasib (*n* = 3, data represented as the mean ± SD, ^**^p < 0.01). (**D**, **E**) Dose-response curves and IC_50_ values for (**D**) the KRAS G12D inhibitor (MRTX1133) and (**E**) the pan-KRAS inhibitor (BI-2865) for organoids with different KRAS mutation statuses.

Sotorasib was the first FDA-approved drug targeting *KRAS* mutations (G12C) in cancer. We determined the IC_50_ values following sotorasib treatment for CWH22, CLM22, two organoids harboring *KRAS* G12D mutations (CRC1-Org and CRC16-Org), and one organoid with wild-type *RAS* (CRC27-Org). The IC_50_ values were 12.07 μM, 10.95 μM, 28.31 μM, 31.37 μM, and 21.49 μM for CWH22, CLM22, CRC1-Org, CRC16-Org, and CRC27-Org, respectively (Figures 4A and 5A). Representative bright-field images of *KRAS* G12D- and G12C-mutated organoids exposed to 10 μM and 20 μM sotorasib are presented in Figure 5B. Based on the *in vitro* cell viability data, the organoids harboring *KRAS* G12C mutations (CWH22 and CLM22) were more sensitive to sotorasib than were those harboring *KRAS* G12D mutations (CRC1-Org, CRC16-Org) or the wild-type RAS (CRC27-Org; Figure 5C). Moreover, a *KRAS* G12D inhibitor (MRTX1133) and a pan-*KRAS* inhibitor (BI-2865) were used to treat all these five organoids, and the dose-response curves and IC_50_ values are presented in Figure 5D, 5E. As expected, *KRAS* G12C-mutated (CWH22, CLM22) and unmutated (CWH27-Org) organoids were less sensitive to MRTX1133 than were *KRAS* G12D-mutated organoids (CRC1-Org and CRC16-Org); however, their sensitivity seemed to be unrelated to the KRAS mutation status when treated with BI-2865. Basic information of CRC1-Org, CRC16-Org, and CRC27-Org is presented in Supplementary Table 1.

### Tumorigenicity and liver metastatic ability of CWH22 and CLM22 *in vivo*

**Figure 6:**
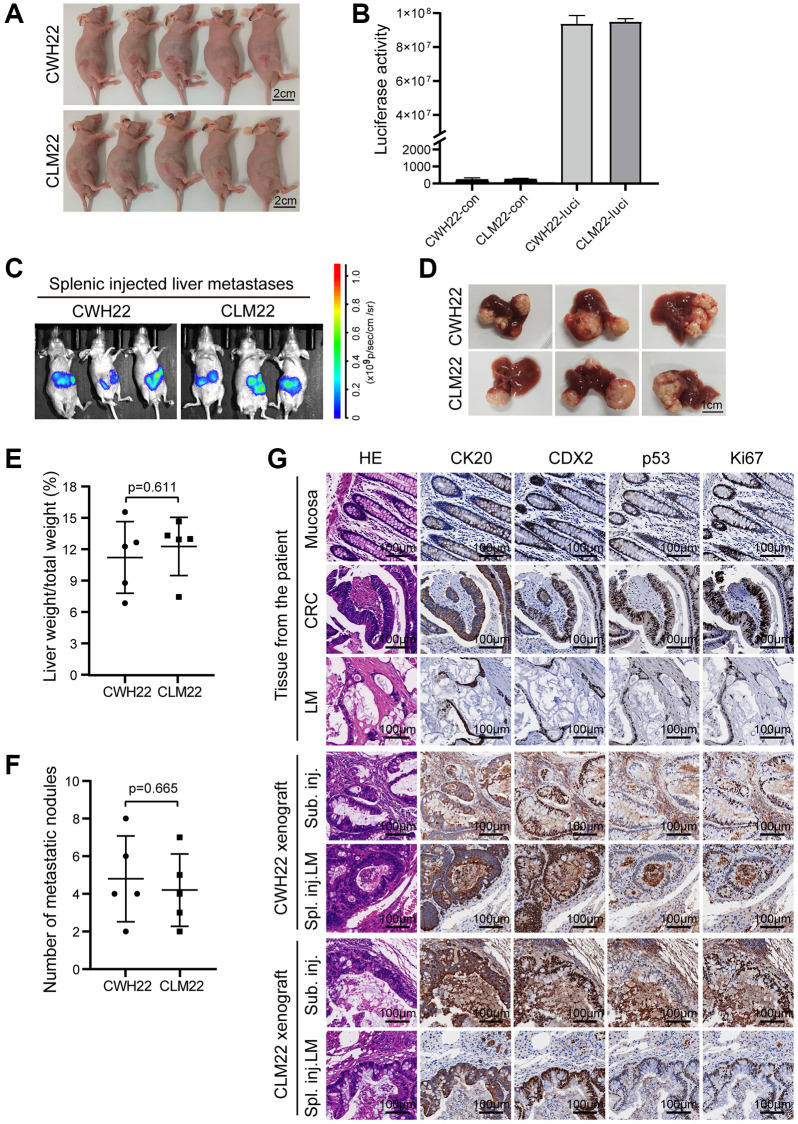
**Tumorigenicity of CWH22 and CLM22 organoids *in vivo*.** (**A**) Images of nude mice bearing CWH22 and CLM22 subcutaneous xenografts (scale bars, 2 cm). (**B**) Luciferase activity tests of CWH22-luci and CLM22-luci (CWH22 and CLM22 with no luciferase expression were tested as control). (**C**) Representative bioluminescence images of mice with liver metastases 42 days after splenic injection. (**D**) Representative pictures of livers with metastatic lesions (on day 45) harvested from mice receiving splenic injections of CWH22 and CLM22 organoid cells (scale bars, 1 cm). (**E**) Quantitative analysis of the percentage of tumor-bearing liver weight to body weight following splenic injection (*n* = 5; data represented as the mean ± SD). (**F**) Quantitative analysis of the metastatic nodule numbers per mouse following splenic injection (*n* = 5; data represented as the mean ± SD). (**G**) H&E morphology and IHC stains of tumor tissues from nude mice in A and B, as well as the normal mucosa and tumor tissues of the patient. Representative images of the expression of CK20, CDX2, Ki67, and p53 are shown (scale bars, 100 μm).

To evaluate the potential of CWH22 and CLM22 organoid cells to grow *in vivo*, these cells were cultured and then injected subcutaneously as organoid structures into nude mice. Images of tumor-bearing mice on day 28 postinjection are presented in Figure 6A, confirming the tumorigenicity of the two organoid cell lines. Then, the organoid cells were transduced with lentivirus supernatants to express luciferase; the organoids stably expressing luciferase were marked as CWH22-luci and CLM22-luci (Figure 6B). Further, the splenic injected liver metastasis models with CWH22-luci and CLM22-luci organoid cells confirmed sound liver metastatic ability of these two organoids, as all injected mice exhibited liver metastases (Figure 6C–6F). Interestingly, the metastatic ability of CWH22 and CLM22 did not significantly differ based on analyses of the percentage of tumor-bearing liver weight to body weight (*p* = 0.611; Figure 6E) and number of liver metastases (*p* = 0.685; Figure 6F). Furthermore, H&E and IHC staining of the xenografts demonstrated that the subcutaneous tumors and transplanted liver metastases induced by CWH22 and CLM22 organoids preserved the moderately differentiated status and the molecular expression status of the corresponding tumor tissues from the patient (Figure 6G). Taken together, these results demonstrated the tumorigenicity and metastatic ability of CWH22 and CLM22 organoids, indicating that the 3D culture system *in vitro* sustained the malignant manifestation and expression status of the corresponding tumors.

### 3D organoids maintain tumor marker expression

Although not every CRC case is associated with increased levels of serum tumor markers (CEA, CA19-9, and CA72-4), the patient from whom CWH22 and CLM22 organoid cell lines were derived had abnormally elevated preoperative CEA and CA19-9 levels (132 ng/mL and 729 U/mL, respectively), which significantly decreased postoperatively (47.05 ng/mL and 151.6 U/mL, respectively). The serum CEA levels of the patient varied with the treatment response and disease progression during the entire treatment period until November 2022 (Supplementary Figure 1D, 1F), as did the trend of CA19-9 levels, consistent with a previous study showing that CEA and CA19-9 levels are clinically relevant for predicting outcomes in patients with metastatic CRC [33].

CEA secretion is an intrinsic feature of some colorectal cancer cell lines, such as LoVo, HCT116, HT-29, T84 [34–36]. To test whether CWH22 and CLM22 organoids maintain the secretion of CEA, as well as CA19-9 and CA72-4, the culture media (incubated for 72 h) were collected on day9 (approximately 1 × 10^5^ cells per well on day 7) and on day15 (approximately 5 × 10^5^ cells per well on day 12) of the 20th passage of CWH22 and CLM22 organoids to assess the levels of these markers; the data are presented in Table 3 (cultured media were collected after incubation with organoids for 72 h), indicating that these two organoid cell lines maintained the expression and secretion characteristics of the original tumors, and that there were no significant differences in the secretion capacity of these tumor markers (CEA, CA19-9 or CA72-4) between CWH22 and CLM22 organoids.

**Table 3:** Tumor marker detection of the patient and the organoid culture medium.

	**Before surgery (Patient serum)**	**After surgery (Patient serum)**	**Out of control (November, 2022; Patient Serum)**	CM^*^	**CWH22 CM (about 1 × 10^5^ cells)^#^**	**CLM22 CM (about 1 × 10^5^ cells)^#^**	**CWH22 CM (about 5 × 10^5^ cells)^#^**	**CLM22 CM (about 5 × 10^5^ cells)^#^**
CEA (≤5 ng/mL)	132↑	47.05↑	165.46↑	<0.5	51.8 ± 3.47↑	61.65 ± 5.17↑	588.87 ± 8.93↑	594.76 ± 8.82↑
CA19-9 (≤34 U/mL)	729↑	151.6↑	2468↑	<0.6	66.08 ± 6.2↑	84.44 ± 10.6↑	630.93 ± 2.16↑	641.29 ± 17.34↑
CA72-4 (<6.9 U/mL)	3.63	1.62	20.55↑	1.57	2.07 ± 0.13	2.09 ± 0.06	51.69 ± 3.15↑	55.56 ± 5.69↑

^*^CM, culture medium; and the total volume of CM is 500 uL per collection. ^#^Cultured for 72 h before collecting the CM, and there are no significant differences between the ability of CWH22 and CLM22 organoids to secrete these tumor markers (*p* > 0.05, independent-samples *T* test).

### Characteristics of adherent CWH22-2D and CLM22-2D cells

**Figure 7:**
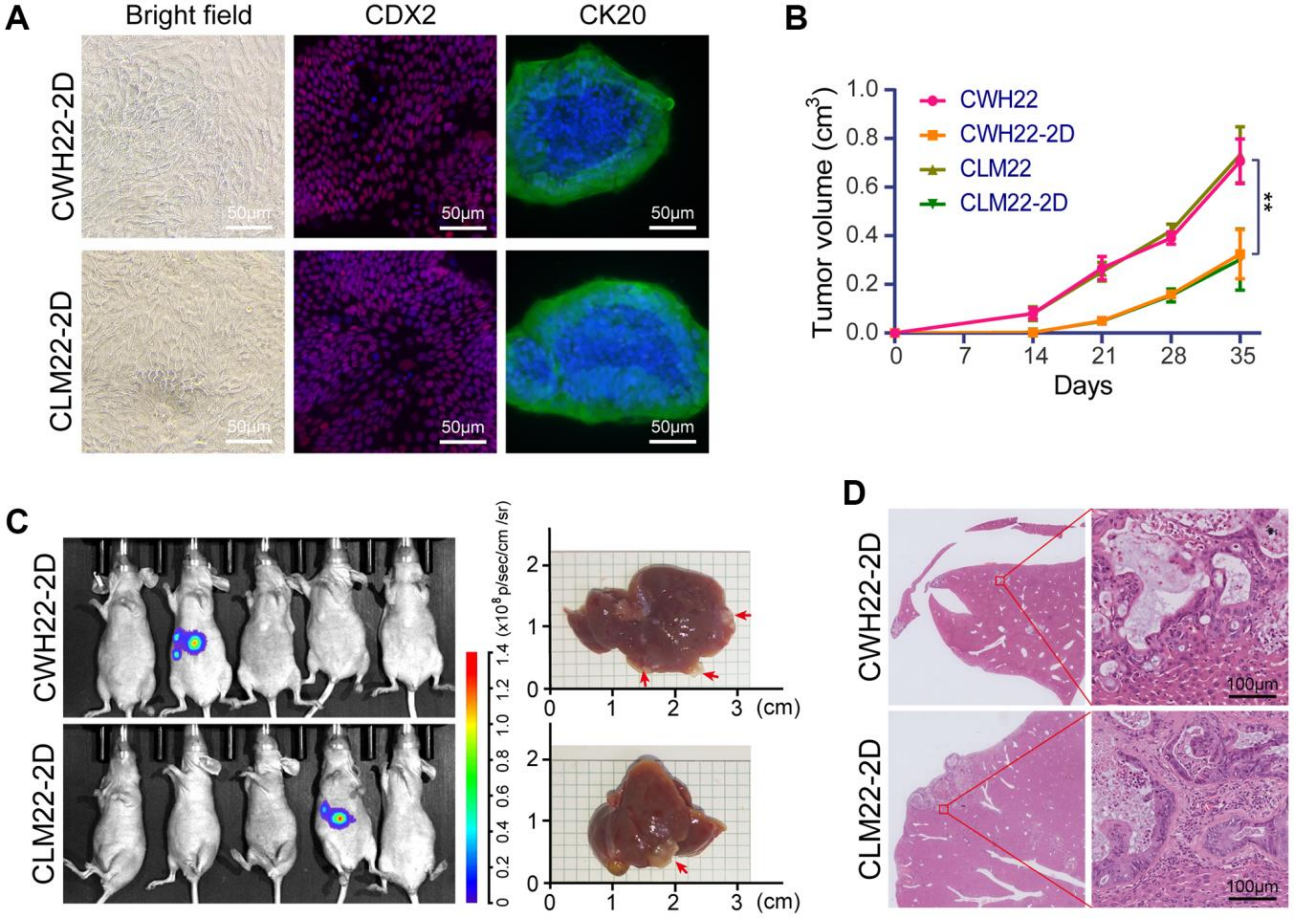
**Tumorigenicity of adherent CWH22-2D and CLM22-2D cells.** (**A**) Bright-field images of CWH22-2D/CLM22-2D cells (left panel); and immunofluorescent staining images of CK20 and CDX2 (middle and right panels) from CWH22-2D/CLM22-2D cells. Scale bars, 50 μm. (**B**) Subcutaneous tumor growth curves were plotted according to the monitored tumor size every seven days for 35 days. The tumors were derived from CWH22-2D/CLM22-2D cells and CWH22/CLM22 organoids. (**C**) Bioluminescence imaging of mice with liver metastases 42 days after splenic injection of CWH22-2D and CLM22-2D cells, and livers with metastatic lesions harvested from mice receiving splenic injections of CWH22-2D and CLM22-2D cells. (**D**) Representative image of the entire liver lobe with metastases, and focal magnification. Scale bars, 100 μm.

CWH22-2D and CLM22-2D adherent cultured cell lines were successfully established from the xenografts derived from CWH22 and CLM22 organoids, after 2 months culture. The bright-field images and the positive immunofluorescent staining of CK20 and CDX2 in CWH22-2D/CLM22-2D cells are presented in Figure 7A. Next, subcutaneous tumor formation assay confirmed xenografts derived from CWH22-2D and CLM22-2D cells grew slower than those derived from CWH22 and CLM22 organoids in nude mice (Figure 7B, *p* < 0.01), and splenic injected liver metastasis formation assays confirmed that CWH22-2D and CLM22-2D cells were not that appropriate to generate liver metastasis models when compared with organoid-derived metastasis models (Figure 6G), as only one mouse tested positive in each group (Figure 7C). H&E staining of CWH22-2D- and CLM22-2D-derived liver metastasis xenografts also displayed a moderately differentiated status (Figure 7D) compared to that of the original tumors, which suggested that differentiation status is an intrinsic property of established cell lines and had no relationship with culture status (2D or 3D) in this study during early use.

### Comparison of CWH22-2D/CLM22-2D with traditional adherent 2D cultured cell lines

Cell lines are important tools for studying tumor pathogenesis, therapies, and drug resistance mechanisms. Their properties in relation to primary tumor characteristics and tumorigenicity in animals indicate their feasibility for research application. Since most CRCs operated on at Tongji Hospital were moderately or moderately to highly differentiated adenocarcinomas, the xenografts formed in immunodeficient mice should reproduce these features to make the models more convincing for further studies.

**Figure 8:**
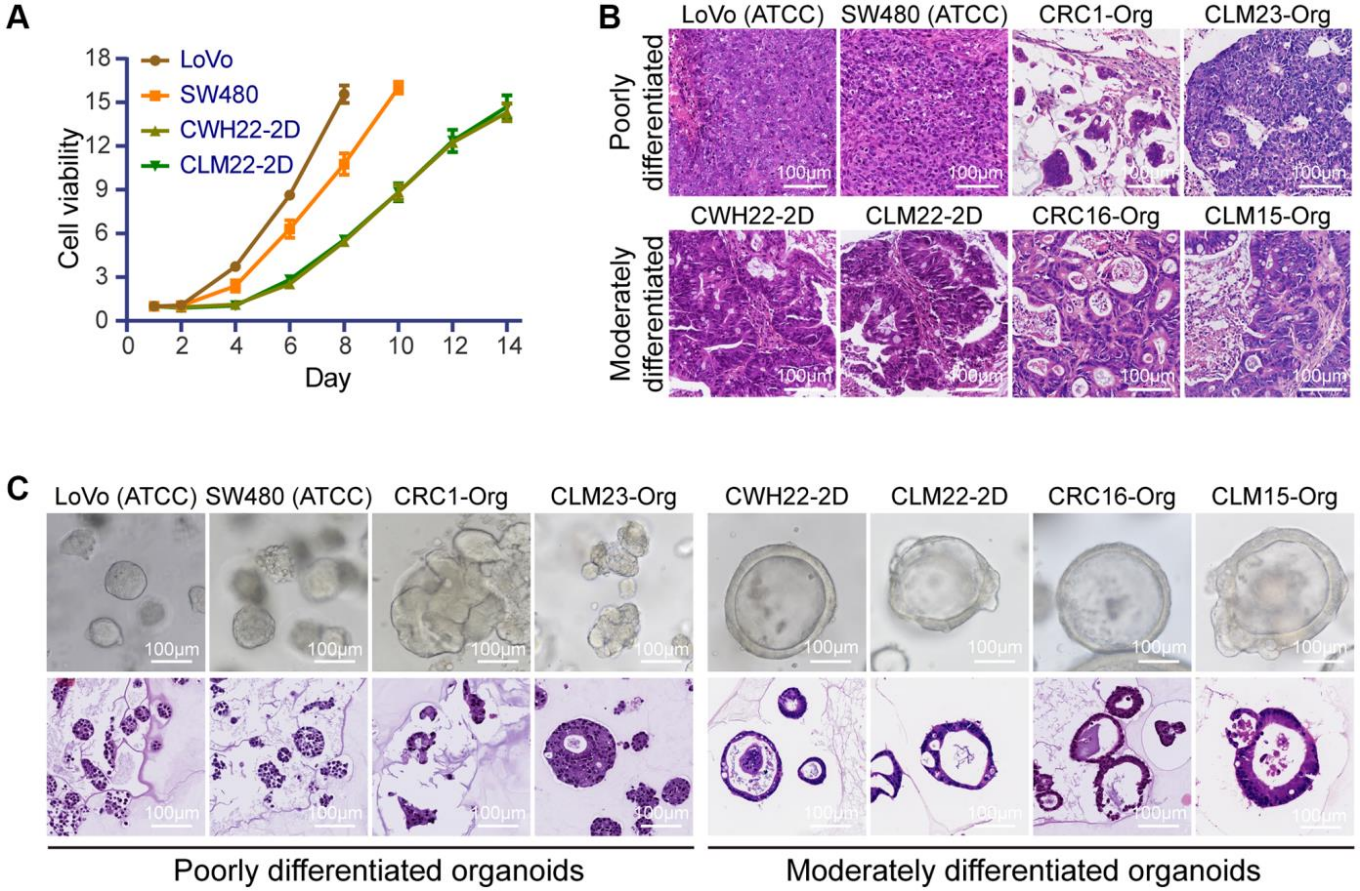
**Differences between CWH22-2D/CLM22-2D and traditional adherent 2D cultured cell lines (LoVo and SW480).** (**A**) Analysis of proliferation rates with CCK-8 in CWH22-2D, CLM22-2D, LoVo, and SW480 cells. (**B**) The differentiation characteristics of subcutaneous neoplasia in nude mice derived from LoVo and SW480 (ATCC), and CWH22 and CLM22 from our culture. Two moderately differentiated (CRC16-Org, CLM15-Org), and two poorly differentiated (CRC1-Org, CLM23-Org) organoid-derived subcutaneous neoplastic tissues were used for comparison. (**C**) Bright-field images and H&E staining of the structures derived from the cells (LoVo, SW480, CRC1-Org, CLM23-Org, CWH22-2D, CLM22-2D, CRC16-Org, and CLM15-Org) cultured in the same 3D culture system. Scale bars, 100 μm.

First, CCK8 assays were conducted to compare the proliferation rates of CWH22-2D, CLM22-2D, LoVo, and SW480 cells, indicated slower growth rates of CWH22-2D and CLM22-2D cells (about 15-fold in the 14 days of culture) compared to that of LoVo and SW480 cells (proliferated about 15-fold in 8–10 days; Figure 8A). Since all these four cell lines possessed well tumorigenicity, we next induced subcutaneous tumor formation in nude mice with LoVo and SW480 cells, and H&E staining exhibited low differentiation status without any tubular or adenoid structures of the xenografts compared with those in the CWH22-2D/CLM22-2D-derived xenografts (Figure 8B). One previous report also presented the same morphological manifestation of the xenografts derived from SW480 in an immunodeficient mouse [37]. Although LoVo cells inoculated into nude mice produced tumor masses with glandular structures 4 decades ago [38], the tumor masses in our study demonstrated the dedifferentiated status of these cells (Figure 8B). Moreover, studies involving H&E and IHC staining of tumor xenografts of other 2D cultured CRC cell lines (from ATCC, such as HCT116, HT-29, Caco-2, and RKO) were searched, and we found that these cell-derived tumors in nude mice presented no evident glandular or adenoid epithelial arrangements in morphology [39–41]. For comparison, H&E staining of subcutaneous tumors derived from two moderately differentiated organoids (CRC16-Org and CLM15-Org) and two poorly differentiated organoids (CRC1-Org and CLM23-Org) are also presented (Figure 8B). Although both CRC1-Org- and CLM23-Org-derived subcutaneous tumors were poorly differentiated, the mucinous lakes and dedifferentiated glandular cavities were preserved, whereas LoVo- and SW480-derived xenografts did not preserve any morphological features associated with the colorectal epithelium (Figure 8B).

Since 3D culture can directly reveal morphology, we conducted 3D organoid cultures with LoVo, SW480, CWH22-2D/CLM22-2D (the 10th generation) cells, and compared with other two moderately differentiated organoids (CRC16-Org, CLM15-Org) and two poorly differentiated organoids (CRC1-Org and CLM23-Org). As indicated, only loose spheroid structures were observed in the bright-field and H&E images of LoVo and SW480 cells, demonstrating a completely dedifferentiated status in these two cell lines compared to other structures (Figure 8C). Thus, both 2D and 3D primary cultures could maintain the differentiation status of the original tumors in our study.

Moreover, the characteristics of several commonly used CRC cell lines from the ATCC and the cell lines established in our laboratory are presented in Table 4, illustrating the origin of these cells, MSH/CIN, CEA secretion, and mutation status of some driver genes. This approach will facilitate the selection and in-depth understanding of these cell lines to different researchers.

**Table 4:** Background information of different CRC cell lines.

**Cell line**	**Patient**	**Disease**	**Derived from**	**Earliest publication**	**MSS/MSI/CIN**	**CEA secretion**	**Chromosome modal no.**	Mutations*								**References**
								**APC**	**BRAF**	**KRAS**	**SMAD4**	**TGFBR2**	**PIK3CA**	**TP53**	**Others**	
CWH22/CWH22-2D	56-year-old female; Asian	Right colon carcinoma	Primary tumor	2023	MSH/CIN	585 ng/3d	50	Frameshift	Wild type	G12C	Q3158D	Wild type	Wild type	Wild type	CDKN1B: Frameshift	——
CLM22/CLM22-2D	56-year-old female; Asian	Right colon carcinoma	Liver metastases	2023	MSH/CIN	604 ng/3d	49	Frameshift	Wild type	G12C	Q3158D	Wild type	Wild type	Wild type	CDKN1B: Frameshift	——
HT-29	44-year-old female; Caucasian	Colon carcinoma	Primary tumor	1972	MSS/CIN	130 ng/24 h	69	E853G; T1556N and Frameshift	V600E	Wild type	Q311G	——	P499T	R273H	——	[42–45]
LoVo	56-year-old male; Caucasian	Colon carcinoma	Left supraclavicularregion metastases	1976	MSI	80 ng/3d	49	R2816Q; R1114G; Frameshift	V600E	G13D, A14V	——	Frameshift	Wild type	Wild type	SMAD2: A292V; FBXW7: R505C	[38, 43, 46]
Caco-2	Caucasian	Colon carcinoma	Primary tumor	1979	MSS/CIN	——	aneuploid	Q1367G	Wild type	Wild type	D351H	——	Wild type	E204	CTNNB1:G245A	[43, 47, 48]
SW480	50-year-old male; Caucasian	Colon carcinoma	Primary tumor	1979	MSS/CIN	21 ng/21d	55	Q1338G	Wild type	G12V	——	——	Wild type	R273H, P309S	——	[43, 49, 50]
SW620	51-year-old male; Caucasian	Colon carcinoma	Lymph node metastasis	1979	MSS/CIN	11 ng/21d	54	Q1338G	Wild type	G12V	——	——	Wild type	R273H, P309S	——	[43, 49, 50]
T84	72-year-old male	Colon carcinoma	Lung metastasis tissue generated subcutaneous xenograft in nude mice	1980	MSS/CIN	——	aneuploid	L1488F and Frameshift	——	G13D	K340N	——	E542K	Splice acceptor mutation	——	[51–53]
HCT116	48-year-old male; Caucasian	Right colon carcinoma	Primary tumor	1981	MSI	76 ng/3d	——	Wild type	Wild type	G13D	——	Frameshift	H1047R	Wild type	CTNNB1: codon deletion; CDKN2A: Frameshift	[35, 43]
RKO	——	Colon carcinoma	Primary tumor	1988	MSI	——	——	Wild type	Wild type	Wild type	——	L452P; K128S and Frameshift	H1047R	Wild type	——	[43, 54, 55]

^*^These mutations are collected from Cellosaurus-Cell line encyclopedia (https://www.cellosaurus.org/).

## DISCUSSION

Cancer cell lines are invaluable biomedical research tools and of great importance for the molecular biology and treatment studies. Effective therapies and preclinical research models for notorious metastatic CRC are lacking. In this study, *in vitro* 3D cultured models and *in vivo* liver metastatic xenografts models from CWH22 and CLM22 organoids (as well as from CWH22-2D/CLM22-2D) may become advanced preclinical models for mechanistic studies, predicting patient response to different therapies, and developing new treatment strategies.

Genomic instability is a hallmark of human cancer that increases the probability of oncogenic events by creating a heterogeneous cell population with an enhanced ability to adapt and evolve. MSI and CIN are the two major alternative genomic instability mechanisms for colorectal carcinogenesis and are detected in approximately 15% and 65–70% of all CRCs, respectively [28, 56]. Several studies have suggested that MSI confers a better prognosis in CRC, whereas CIN is associated with a worse prognosis [56]. In the present study, CIN was the primary cause of genomic instability in CWH22/CLM22 and corresponding tumors. In clinical practice, the patient was first sensitive to the eight cycles of treatment with mFOLFOX + bevacizumab; however, the patient became resistant to subsequent capecitabine, mFOLFIRI + bevacizumab, and mXELIRI + bevacizumab treatments one year later (Supplementary Figure 1D–1F), and died with an overall survival of 27 months, confirming the poor prognosis in this metastatic case with CIN.

*KRAS* mutations are found in ~40% of CRCs, with *KRAS* G12C mutation occurring in 1–3% of CRCs [29]. A phase I trial demonstrated that sotorasib provided clinical benefit for *KRAS* G12C-mutated non-small cell lung cancers with an objective response rate of 32.2%, compared to only 7.1% for CRC, in patients with advanced solid tumors [57]. According to a phase II clinical trial, the objective response rate was 9.7%, the disease control rate was 82.3%, and progression-free survival was four months, indicating modest sotorasib benefits in CRC patients [29, 58]. According to our *in vitro* experiments, CWH22 and CLM22 harboring *KRAS* G12C mutations exhibited a more favorable response to sotorasib than did those organoids harboring *KRAS* G12D mutations or wild-type RAS (CRC1-Org, CRC16-Org, and CRC27-Org; Figure 5C); however, sotorasib appeared to be less effective than 5-Fu, possibly due to the heterozygosity of *KRAS* G12C mutations in CWH22 and CLM22. Since previous clinical trials have presented that acquired resistance to *KRAS* G12C inhibition is associated with acquired *KRAS* alterations and resistance bypass mechanisms [59, 60], appropriate combined treatment strategies with sotorasib warrant further exploration. Thus, CWH22/CLM22 cells will be useful preclinical models for further research and represent the subgroup of CRC patients (1–3%) with KRAS G12C mutations.

In addition, CWH22 and CLM22 organoids appeared to respond poorly to both the KRAS G12D inhibitor (MTRX1123) and the pan-*KRAS* inhibitor (BI-2865), while CRC1-Org was sensitive to both inhibitors (Figure 5D, 5E). On the contrary, although CRC16-Org possessed the same KRAS mutation status as CRC1-Org, it was intrinsically resistant to these targeted inhibitors (Figure 5D, 5E). Thus, we speculate that one single mutation could not fully determine the sensitivity of a certain corresponding targeted therapy due to the heterogeneity of different cases. Moreover, several previous studies about genomics to guide cancer therapies demonstrated that less than 7% of patients were estimated to benefit from such precision oncology in 2018 [31]. Thus, drug testing on the patient’s tumor-derived organoids offers a complementary and effective approach and might deserve wide application in personalized treatments [61].

Despite growing insights into CRC biology and many therapeutic improvements, preclinical models for CRC and metastatic CRC *in vivo* are still indispensable for the development of new treatment approaches. The animal models for CRC based on injection of common widely used 2D CRC cell lines lack genetic diversity and proper differentiation due to decades of use, as presented in our research (Figure 8B) and other studies [39–41]. Genetically engineered mouse models exhibit CRC pathogenesis dependent on the altered molecular pathways, but lack metastasis and tumor heterogeneity, and are largely restricted to the small intestine [62, 63]. In contrast, organoid-derived mouse models maintained the differentiation status and metastatic ability of the original tumors (Figure 6C, 6G), representing the most reliable models for preclinical studies.

Although encouraged by the known characteristics of these two organoids, we are aware of several limitations to our research. First, though liver metastasis models have been established, these are not orthotopic liver metastatic models, which require longer modeling cycles (more than 120 days, and metastatic rates <20% in pre-experiment tests). Thus, the spleen-injected liver metastatic model was the preferred modeling selection. Second, all animal models used were established in immunodeficient mice, which are unsuitable for immunology-related studies *in vivo*; humanized mice could be proper models for tumor organoid-related immune studies *in vivo* [64]. Third, considering that both CWH22 and CLM22 organoids exhibited strong metastatic ability *in vivo* (Figure 6C–6F), the study of the difference between these two organoids is not suitable for the study of metastatic mechanisms, unless compared to other nonmetastatic organoids. While considering the difference presented in Figures 6C and 7C, the different metastatic rates between CWH22/CLM22 organoid cells and CWH22-2D/CLM22-2D cells *in vivo* are possibly due to the difference in stem cell abundance, as serum-free culture medium sustains greater numbers of stem cells. Furthermore, although CWH22 and CLM22 came from the primary and metastatic lesions of the same CRC patient, none of the tests in this study showed any differences between them, which could be presented in epigenetic or otherwise, pending further research. Beyond these limitations, due to the degradation of tissue RNAs during sample collection and preservation, it was not possible to conduct RNA analysis among tumor tissues and organoids.

Since *APC*, *KRAS*, *SMAD4,* and *CDKN1B* are \commonly mutated genes in CRC and mCRC, and *KMT2C* mutations have been associated with metastasis and poor survival in patients with lung cancer [65, 66], CWH22 and CLM22 are representative CRC cell lines with mutated *APC*/*KRAS*/*SMAD4*/*CDKN1B/KMT2C* genes and wild-type *TP53* and *PIK3CA*. They contribute to the diversity of CRC cell lines, and to the exploration of the molecular mechanisms underlying CRC pathogenesis and metastasis, facilitating the screening and evaluation of antitumor drugs in CRC/CLM studies, and rendering preclinical research more reliable.

3D organoid culture, despite the high cost, can better maintain the morphology and differentiation status of the corresponding tumor *in vitro* (Figure 2), as well as the metastatic ability *in vivo* (Figures 6C and 7C); the cost of 2D culture is comparably more acceptable than that of other methods, but selection pressure is exerted on cells during culture and passage due to the large difference from the growth environment *in vivo*. At early passages, 2D cultured cells can maintain the differentiation status when injected into immunodeficient mice (Figure 8); however, long-term cultured 2D cell lines present to be homogenous, lose intratumor heterogeneity, and exhibit low genetic diversity due to high passage and selective pressure [67]. Therefore, regardless of the cost, 3D organoids are better than early passaged 2D adherent cell lines, and then the traditionally long-term passaged 2D cell lines.

In summary, we report paired organoids (CWH22/CLM22) and the corresponding adherent cell lines (CWH22-2D/CLM22-2D) with sound background information and potential applications. By comparison with other CRC cell lines (Table 4), researchers who are interested in CRC, metastatic CRC, and the corresponding treatments could obtain these cell lines from the CCTCC (No. C202218, C202219; WDPP011, WDPP010) or from our laboratory upon reasonable request.

## MATERIALS AND METHODS

### Clinical history and patient characteristics

In this study, the presented paired organoids were established from the tumor tissues of a patient with CRC and synchronous liver metastasis. Both the organoids and the corresponding tumor tissues were subjected to WES and histopathological analysis to determine the concordance between tumor organoids and corresponding tumors, and organoids were used for further study. Figure 1 presents an overview of the procedures.

The tissue donor, a 65-year-old woman, was admitted to the Hepatic Surgery Centre of Tongji Hospital in January 2021 with a two-week history of weakness after the discovery of a liver-occupying lesion during a physical examination. Computed tomography (CT) and magnetic resonance imaging (MRI) scans of the liver revealed an 89 × 65 mm lesion in the left liver lobe (Supplementary Figure 1A). Routine blood examination showed moderate anemia (hemoglobin level, 74 g/L). A colonoscopy revealed a space-occupying lesion in the right colon (Supplementary Figure 1B). Laparoscopic resections of the lesions from the right colon and left liver lobe were performed. The histological diagnosis revealed moderately differentiated right colon invasive adenocarcinoma with liver metastasis at stage IV A (T3N1cM1a; Supplementary Figure 1C). Several small liver metastases occurred during the recovery from the surgery, and on day 45 after surgery, the patient started treatments with mFOLFOX + bevacizumab. The small metastatic lesions shrank and were stable during the eight treatment cycles. Thereafter, capecitabine and bevacizumab were used as maintenance treatments, and the lesion appeared stable for an additional seven months. However, the disease relapsed 14 months later, and became uncontrollable in November 2022. The entire treatment strategies until November 2022 and treatment responses are presented in Supplementary Figure 1D. Supplementary Figure 1E, 1F present the treatment response of the liver lesions (MRI images) and changes in the serum carcinoembryonic antigen (CEA) concentration during the treatment.

### Tissue sampling

Samples were obtained from the Hepatic Surgery Centre, Tongji Hospital, Huazhong University of Science and Technology (Wuhan, China). The collected CRC and CLM samples were immediately divided into three parts. One part was immersed in DMEM, transported on ice to the laboratory for tumor cell isolation, and cultured within 1–3 h. The remaining parts were placed in liquid nitrogen and 4% paraformaldehyde (Sigma-Aldrich, P6148) immediately after collection for molecular and histopathological examinations.

### Tumor cell isolation and 3D organoid culture

For the derivation of paired tumoroids, freshly resected CRC or CLM samples were processed as previously reported [22]. The surface mucus and necrotic tissue of the tumor sample were removed before washing with ice-cold phosphate-buffered saline (PBS), and the tissue was soaked in active iodine for 1 min. Subsequently, the samples were rinsed thrice with PBS, penicillin/streptomycin, and PBS successively for each step. Then, the tissue (~0.5 cm^3^) was cut into 0.5 mm pieces, digested in 50% TrypLE™ Express (Gibco, 12605028)/50% DMEM at 25°C for 15 min (the suspension was mixed thoroughly at 7 and 14 min), and neutralized with the same volume of Ca^2+^-containing Hanks’ balanced salt solution (HBSS). The cell suspension was allowed to settle for 3–5 min, after which the supernatant was collected, centrifuged (500 × g), and washed twice using centrifugation at different speeds (140 × g and 200 × g) to remove cell debris and collect tumor cells. The pellet was then washed twice in advanced DMEM/F12, and cells were counted and mixed with growth factor-reduced BME (RD, 3533). In a prewarmed 24-well plate, 8,000–10,000 cells were seeded per well with 30 μL BME. After BME solidification following incubation at 37°C for 20–30 min, 600 μL of tumoroid-specific culture medium (Table 5) was added, and the plate was incubated at 37°C with 5% CO_2_. The medium was completely refreshed every 3–5 days.

**Table 5:** Overview of the culture medium components.

**Reagent**	**Source**	**Cat. No.**	**Concentration**
Advanced DMEM/F12	Gibco	12634010	–
B27	Gibco	12587010	1 ×
HEPES buffer	Gibco	15630106	10 mM
GlutMAX	Gibco	35050-61	1 ×
N-Acetyl-L-Cysteine	Sigma	A7250-50G	1 mM
Nicotinamide	Sigma	N0636-100G	4 mM
Prostaglandin E2	Tocris	2296-10	1 μM
Gastrin I human	Sigma	G9145-5MG	10 nM
FGF-b	Peprotech	100-18B-1000	20 ng/mL
FGF-10	Peprotech	100-18B-1000	20 ng/mL
Y27632	Stemcell	72302	1 μM^*^

^*^Only used in the first two days of each passage.

During the first two passages, only half of the medium was changed each time. Organoids were passaged when they reached diameters of 100–700 μm. After washing with PBS, both the organoids and BME fractions were mechanically pipetted using 250 μL TrypLE™ Express per well. Organoids were digested into single cells within 10–14 min. Following neutralization with the same volume of Ca^2+^-containing HBSS, the cells were washed with DMEM, counted, seeded at 8000 cells/30 μL BME, overlaid with organoid-specific culture medium, and cultured as described above.

After subsequent passages, all stably passaged organoids were viably frozen as single cells in culture medium with 10% dimethyl sulfoxide and stored at −80°C, and cryovials were transferred to a nitrogen tank the next day—for long-term storage. Organoid cultures were checked monthly for mycoplasma contamination using a MycoBlue Mycoplasma Detector Kit (Vazyme, D101-01/02, China). The organoid cell lines derived from the resected CRC and CLM tissues were named CWH22 and CLM22, respectively. Both were cultured and passaged using the same protocols.

### Immunohistochemistry and immunofluorescence assays

Tissues from the patient and tumor-burdened nude mice were embedded in paraffin. All hematoxylin and eosin (H&E) and immunohistochemistry (IHC) staining procedures, and subsequent histological diagnoses were performed in the Pathology Department of Tongji Hospital, as previously reported [68]. The antibodies used for IHC were as follows: CDX2 (Cat. No. ZA-0520 R), CK20 (Cat. No. ZA-0574 R), MLH1 (Cat. No. ZM-0154), PMS2 (Cat. No. ZA-0542 R), MSH2 (Cat. No. ZA-0622 R), MSH6 (Cat. No. ZA-0541 R), Her-2 (Cat. No. ZA-0641 R), Ki67 (Cat. No. ZM-0166), p53 (Cat. No. ZM-0408), and Villin (Cat. No. ZA-0575 R; all from ZSGB-BIO).

The cultured organoids (usually 4–6 wells) were harvested and fixed in 4% paraformaldehyde overnight, then, wrapped in Histogel (Epredia, USA, HG-4000-012), dehydrated, and embedded in paraffin blocks. H&E and IHC were performed on the organoids as indicated above for tumor tissues. For immunofluorescence staining, Ki67 (CST 9449) and CK20 (Abcam, ab76126) were detected at the same time for CWH22 and CLM22 organoids. Images of immunofluorescence staining were obtained using confocal laser-scanning microscopy on a Nikon Digital ECLIPSE C1 system (Nikon Corporation, Japan).

### Authentication of CWH22/CLM22 organoids

For authentication, DNA from CWH22 and CLM22 organoid cells and normal mucosa tissue from the patient were isolated using an AxyPrep Multisource Genomic DNA Miniprep Kit (Axygen, Corning, Inc., USA), and 21 STR loci were examined and compared with the corresponding STR profile of the normal tissue at the CCTCC. After bacterial and mycoplasma contamination tests were confirmed to be negative, the CWH22 and CLM22 organoid cell lines were deposited at the CCTCC (No. C202218, C202219).

### WES analysis of organoids and tumor tissues

CWH22 and CLM22 organoids were harvested for DNA extraction after being cultured for 12 days. DNA from cultured organoids and liquid nitrogen-frozen tissues (normal mucosa, CRC, and LM) of the corresponding patient was extracted using a Zymo Quick-DNA Microprep kit (Zymo Research, USA #D2030) according to the manufacturer’s protocol. Library construction and whole-exome capture of genomic DNA were performed using the SureSelectXT Human All Exon V6 (Agilent, USA) and MGIEasy Exome Universal Library Prep Set-V1.0. The captured DNA was sequenced using a MGISEQ 2000 platform (BGI-Shenzhen, China) with 150 bp paired-end sequencing. A total of >0.4 μg DNA per sample was used as input. The effective sequencing depth was above 100× (10 G) per sample. The human genome data of Hg19 from the UCSC Genome Browser (http://genome.ucsc.edu/) was used as a reference, and common mutated genes in CRC and LM associated with CRC were analyzed for each sample.

### Chromosome karyotype analysis

Metaphase chromosome preparations were conducted by treating organoids in the exponential growth phase (usually at 7–10 days) with 0.6 μg/mL colchicine for 5 h. Then, enzymatic dissociation was performed with TrypLE™ Express to disperse the organoids into single cell suspensions. The suspension was centrifuged at 1,000 × g for 6 min. Preheated 0.075 mM KCl hypotonic solution was added and incubated for 15 min at 37°C, then fixed with 3:1 methanol-acetic acid (v: v; 1 min). Subsequently, the pellets were fixed twice at room temperature for 10 min, and 500 μL stationary liquid was added to resuspend and collect the cells. Afterward, cell droplets were spotted on clean, precooled microscope glass slides. The slides were immediately heated over an alcohol lamp for 1 s to allow the chromosomes to spread out. Chromosome specimens were stained with Giemsa (#BA4219, Baso Diagnostics, China) for 10 min at 25–28°C according to the manufacturer’s instructions, and the chromosomes of M phase cells were counted using an oil immersion lens (100×) under a microscope (Nikon, Tokyo, Japan). Karyotypes were determined by arranging all the photographed metaphases. The chromosomes were classified according to the International System for Human Cytogenetic Nomenclature [69].

### Organoid proliferation assay

When the organoids (CWH22 and CLM22) entered the logarithmic growth phase between days 9–11 of each passage, they were mechanically and enzymatically dissociated into single cells by incubation in TrypLE™ Express for 10–14 min and resuspended in BME at a concentration of 3 × 10^5^/mL separately on ice. To assess proliferation, the suspensions were seeded onto pre-warmed 96-well plates (Greiner Bio-one, 655090) in triplicate at 3 × 10^3^ cells per well (10 μL/well) on days 1, 4, 7, 10, and 14 and maintained in complete culture medium (100 μL/well) for 14 days. Quantification of cell viability was performed by replacing the culture medium with 50 μL of CellTiter-Glo 3D (#G9681, Promega, USA) mixed with 50 μL of culture medium on an Infinite 200 PRO plate reader (Tecan Life Sciences) according to the manufacturer’s instructions. Organoid proliferation rates were determined, and graphs were drawn using GraphPad Prism 8 (GraphPad Software, Inc.).

### 2D-cultured cell proliferation tests

The establishment of CWH22-2D and CLM22-2D was done from the subcutaneous tumors derived from 3D organoids CWH22 and CLM22. The tissues were digested into single cells and clusters as presented above and then seeded in culture plates with high-glucose DMEM containing 10% FBS. When cultured for about 2 months and passaged more than 4 times, the growth rates and cellular morphology became stable, allowing further analysis.

Cells (LoVo, SW480, CWH22-2D and CLM22-2D) were seeded onto 96-well plates at 4 × 10^3^ cells per well in 100 μL suspensions in triplicate per day and maintained in complete culture medium. The cell proliferation rate was measured using the Cell Counting Kit-8 assay (CCK-8; Cat. No. KJ800; Dojindo Laboratories, Kumamoto, Japan) according to the manufacturer’s instructions as previously reported [68].

### Tumor marker detection

Clinically, serum tumor markers (CEA, CA19-9, and CA72-4) were detected before and after surgery, and the CEA and CA19-9 levels of the patient (from whom CWH22 and CLM22 were derived) were abnormal. The culture medium of the CRC/CLM-derived organoids at passage 20 (incubated for 3 days, 500 μL) was collected for tumor marker detection. All measurements were conducted in the Laboratory Medicine of Tongji Hospital under the same conditions.

### IC_50_ tests

In the present study, 5-fluorouracil (5-Fu; Cat. No. HY-90006), oxaliplatin (Cat. No. HY-17371), SN-38 (Cat. No. HY-13704; an active metabolite of irinotecan), VP-16 (Cat. No. HY-13629), regorafenib (Cat. No. HY-10331), and sotorasib (Cat. No. HY-114277) were selected for drug sensitivity tests (all purchased from MedChemExpress, USA). We chose 9–12 different concentrations of each drug to test, based on the results of preliminary experiments. Organoids were mechanically and enzymatically dissociated into single cells, resuspended in BME at 3 × 10^5^/mL, and seeded at 10 μL per well in prewarmed 96-well plates (Greiner Bio-one, 655090). The cells were maintained in complete culture medium (100 μL/well) as described above. Organoids were considered ready for IC_50_ tests when they reached 100 μm in diameter, at which point the culture medium was replaced with media containing different drug concentrations. Those organoids were exposed to various drugs for six days, and the culture medium was changed on the third day. Subsequently, cell viability was assessed using the CellTiter-Glo 3D Cell viability assay (#G9683, Promega, USA ), as presented above. Dose-response curves were plotted, and the IC_50_ values were calculated using GraphPad Prism 8.

MRTX1133 (a *KRAS* G12D inhibitor; Cat. No. HY-134813), BI-2865 (a pan-*KRAS* inhibitor; Cat. No. HY-153724), and sotorasib (a *KRAS* G12C inhibitor) were also introduced into the media containing the CWH22/CLM22 organoids (*KRAS* G12C mutated), CRC1-Org/CRC16-Org (*KRAS* G12D mutated), and CRC27-Org (wild-type *RAS*) to draw dose-response curves, calculate IC_50_ values, and determine the sensitivity of KRAS targeted therapies *in vitro*.

### Construction of organoids stably expressing luciferase

The luciferase gene was cloned into the pLVX-IRES-Neo plasmid using XhoI/EcoRI restriction sites to produce a luciferase-carrying lentivirus. Lentivirus supernatant was collected as previously reported [70]. To establish organoids stably expressing luciferase (CWH22-luci and CLM22-luci), single-cell suspensions of the CWH22 and CLM22 organoid cells (the second generation) were seeded on the surface of presolidified BME in 96-well plates (30 μL diluted BME, 5000 cells/well, 10 wells/organoid line). Approximately 48–72 h later, the lentivirus supernatant was used to transduce each organoid (nine wells for transduction, one well for selection control); 5 μg/mL polybrene was added to improve the transfection efficiency and was replaced with organoid culture medium after 12 h. At 48 h post-transduction, the culture medium was replaced with selection medium containing 500 μg/mL G418 for at least seven days to kill any cells that were not transfected. Once the cells in the control well died thoroughly, and the luciferase-expressing cells were grown into organoid structures 100 μm in diameter, the organoids were harvested and enzymatically dissociated into single cells using TrypLE™ Express for 10–14 min. Cells were subsequently passaged as described above. On the 10th day from the first passage post-transfection, one well of each organoid (CWH22-luci and CLM22-luci) was harvested and tested separately for luciferase activity on a Gloma 20/20 luminometer (Promega, USA) with 20 μL *Vivo* GloTM Luciferin reagent (10 mg/mL, Promega, P1403, USA), un-transfected organoids (CWH22-con and CLM22-con) were also harvested for control test.

### Determination of tumorigenicity

A total of 20 BALB/c athymic nude mice were housed under specific pathogen-free conditions in a temperature- and humidity-controlled environment (room temperature, 20–26°C; humidity, 40–60%; 12-h light/dark cycle; free access to food and water). First, CWH22 and CLM22 organoids (the third generation) were harvested with BME and injected subcutaneously into the right hip of each mouse (1.5 × 10^6^ cells/mouse, five mice/organoid) to test tumorigenicity. On day 28, the mice were tested for the tumor–burdened status, and the subcutaneous tumors were subjected to histopathological analysis.

Next, the liver metastatic capacities of CWH22 and CLM22 organoids were tested *in vivo*. Ten female mice were divided into two groups, with five mice per organoid. Single-cell suspensions containing 1 × 10^6^ cells (CWH22-luci, CLM22-luci, total fifth generation) were injected into each anesthetized mouse spleen as described previously [71]. At the onset of weight loss, bioluminescence imaging indicated definite liver metastases in each mouse. On day 45, the mice were euthanized with an overdose of intraperitoneal pentobarbital sodium, and metastatic livers were harvested, measured, fixed in 4% paraformaldehyde overnight at 25–28°C, and embedded in paraffin.

The procedures for detecting the tumorigenicity and metastatic ability of CWH22-2D and CLM22-2D cells were the same as those described above for 3D organoids (1.5 × 10^6^ cells/mouse for subcutaneous injection, 1 × 10^6^ cells/mouse for spleen injection).

### Statistical analysis

Statistical analyses were performed using SPSS 19 and GraphPad Prism 8. Independent sample *t*-test and one-way analysis of variance were used as appropriate. A *p*-value < 0.05 was considered to indicate statistical significance.

### Availability of data and materials

The datasets used and analyzed in the current study are available from the corresponding author upon reasonable request. The WES dataset generated and analyzed in the present study is available in the NCBI repository (https://www.ncbi.nlm.nih.gov/bioproject/PRJNA902102). All the authors had access to the study data and reviewed and approved the final manuscript.

## Supplementary Materials

Supplementary Figure 1

Supplementary Table 1

Supplementary Video 1

Supplementary Video 2

## References

[1] Sung H, Ferlay J, Siegel RL, Laversanne M, Soerjomataram I, Jemal A, Bray F. Global Cancer Statistics 2020: GLOBOCAN Estimates of Incidence and Mortality Worldwide for 36 Cancers in 185 Countries. CA Cancer J Clin. 2021; 71:209–49. 10.3322/caac.21660 33538338

[2] Siegel RL, Miller KD, Fuchs HE, Jemal A. Cancer Statistics, 2021. CA Cancer J Clin. 2021; 71:7–33. 10.3322/caac.21654 33433946

[3] Siegel RL, Miller KD, Goding Sauer A, Fedewa SA, Butterly LF, Anderson JC, Cercek A, Smith RA, Jemal A. Colorectal cancer statistics, 2020. CA Cancer J Clin. 2020; 70:145–64. 10.3322/caac.21601 32133645

[4] Chua TC, Saxena A, Chu F, Zhao J, Morris DL. Predictors of cure after hepatic resection of colorectal liver metastases: an analysis of actual 5- and 10-year survivors. J Surg Oncol. 2011; 103:796–800. 10.1002/jso.21864 21246567

[5] Ren L, Zhu D, Benson AB 3rd, Nordlinger B, Koehne CH, Delaney CP, Kerr D, Lenz HJ, Fan J, Wang J, Gu J, Li J, Shen L, et al, and SINCE (Shanghai International Consensus Expert Group on Colorectal Liver Metastases) Group. Shanghai international consensus on diagnosis and comprehensive treatment of colorectal liver metastases (version 2019). Eur J Surg Oncol. 2020; 46:955–66. 10.1016/j.ejso.2020.02.019 32147426

[6] Rees M, Tekkis PP, Welsh FK, O'Rourke T, John TG. Evaluation of long-term survival after hepatic resection for metastatic colorectal cancer: a multifactorial model of 929 patients. Ann Surg. 2008; 247:125–35. 10.1097/SLA.0b013e31815aa2c2 18156932

[7] Van Cutsem E, Cervantes A, Adam R, Sobrero A, Van Krieken JH, Aderka D, Aranda Aguilar E, Bardelli A, Benson A, Bodoky G, Ciardiello F, D'Hoore A, Diaz-Rubio E, et al. ESMO consensus guidelines for the management of patients with metastatic colorectal cancer. Ann Oncol. 2016; 27:1386–422. 10.1093/annonc/mdw235 27380959

[8] Huang Y, Liu Y, Zheng C, Shen C. Investigation of Cross-Contamination and Misidentification of 278 Widely Used Tumor Cell Lines. PLoS One. 2017; 12:e0170384. 10.1371/journal.pone.0170384 28107433 PMC5249119

[9] Capes-Davis A, Theodosopoulos G, Atkin I, Drexler HG, Kohara A, MacLeod RA, Masters JR, Nakamura Y, Reid YA, Reddel RR, Freshney RI. Check your cultures! A list of cross-contaminated or misidentified cell lines. Int J Cancer. 2010; 127:1–8. 10.1002/ijc.25242 20143388

[10] Yates LR, Campbell PJ. Evolution of the cancer genome. Nat Rev Genet. 2012; 13:795–806. 10.1038/nrg3317 23044827 PMC3666082

[11] Drost J, Clevers H. Organoids in cancer research. Nat Rev Cancer. 2018; 18:407–18. 10.1038/s41568-018-0007-6 29692415

[12] Yao Y, Xu X, Yang L, Zhu J, Wan J, Shen L, Xia F, Fu G, Deng Y, Pan M, Guo Q, Gao X, Li Y, et al. Patient-Derived Organoids Predict Chemoradiation Responses of Locally Advanced Rectal Cancer. Cell Stem Cell. 2020; 26:17–26.e6. 10.1016/j.stem.2019.10.010 31761724

[13] Sachs N, de Ligt J, Kopper O, Gogola E, Bounova G, Weeber F, Balgobind AV, Wind K, Gracanin A, Begthel H, Korving J, van Boxtel R, Duarte AA, et al. A Living Biobank of Breast Cancer Organoids Captures Disease Heterogeneity. Cell. 2018; 172:373–86.e10. 10.1016/j.cell.2017.11.010 29224780

[14] Lau HCH, Kranenburg O, Xiao H, Yu J. Organoid models of gastrointestinal cancers in basic and translational research. Nat Rev Gastroenterol Hepatol. 2020; 17:203–22. 10.1038/s41575-019-0255-2 32099092

[15] Mo S, Tang P, Luo W, Zhang L, Li Y, Hu X, Ma X, Chen Y, Bao Y, He X, Fu G, Xu X, Rao X, et al. Patient-Derived Organoids from Colorectal Cancer with Paired Liver Metastasis Reveal Tumor Heterogeneity and Predict Response to Chemotherapy. Adv Sci (Weinh). 2022; 9:e2204097. 10.1002/advs.202204097 36058001 PMC9631073

[16] Ganesh K, Wu C, O'Rourke KP, Szeglin BC, Zheng Y, Sauvé CG, Adileh M, Wasserman I, Marco MR, Kim AS, Shady M, Sanchez-Vega F, Karthaus WR, et al. A rectal cancer organoid platform to study individual responses to chemoradiation. Nat Med. 2019; 25:1607–14. 10.1038/s41591-019-0584-2 31591597 PMC7385919

[17] Ooft SN, Weeber F, Dijkstra KK, McLean CM, Kaing S, van Werkhoven E, Schipper L, Hoes L, Vis DJ, van de Haar J, Prevoo W, Snaebjornsson P, van der Velden D, et al. Patient-derived organoids can predict response to chemotherapy in metastatic colorectal cancer patients. Sci Transl Med. 2019; 11:eaay2574. 10.1126/scitranslmed.aay2574 31597751

[18] Bruun J, Kryeziu K, Eide PW, Moosavi SH, Eilertsen IA, Langerud J, Røsok B, Totland MZ, Brunsell TH, Pellinen T, Saarela J, Bergsland CH, Palmer HG, et al. Patient-Derived Organoids from Multiple Colorectal Cancer Liver Metastases Reveal Moderate Intra-patient Pharmacotranscriptomic Heterogeneity. Clin Cancer Res. 2020; 26:4107–19. 10.1158/1078-0432.CCR-19-3637 32299813

[19] Weeber F, van de Wetering M, Hoogstraat M, Dijkstra KK, Krijgsman O, Kuilman T, Gadellaa-van Hooijdonk CG, van der Velden DL, Peeper DS, Cuppen EP, Vries RG, Clevers H, Voest EE. Preserved genetic diversity in organoids cultured from biopsies of human colorectal cancer metastases. Proc Natl Acad Sci U S A. 2015; 112:13308–11. 10.1073/pnas.1516689112 26460009 PMC4629330

[20] Fusenig NE, Capes-Davis A, Bianchini F, Sundell S, Lichter P. The need for a worldwide consensus for cell line authentication: Experience implementing a mandatory requirement at the International Journal of Cancer. PLoS Biol. 2017; 15:e2001438. 10.1371/journal.pbio.2001438 28414712 PMC5393552

[21] Souren NY, Fusenig NE, Heck S, Dirks WG, Capes-Davis A, Bianchini F, Plass C. Cell line authentication: a necessity for reproducible biomedical research. EMBO J. 2022; 41:e111307. 10.15252/embj.2022111307 35758134 PMC9289526

[22] van de Wetering M, Francies HE, Francis JM, Bounova G, Iorio F, Pronk A, van Houdt W, van Gorp J, Taylor-Weiner A, Kester L, McLaren-Douglas A, Blokker J, Jaksani S, et al. Prospective derivation of a living organoid biobank of colorectal cancer patients. Cell. 2015; 161:933–45. 10.1016/j.cell.2015.03.053 25957691 PMC6428276

[23] Broutier L, Mastrogiovanni G, Verstegen MM, Francies HE, Gavarró LM, Bradshaw CR, Allen GE, Arnes-Benito R, Sidorova O, Gaspersz MP, Georgakopoulos N, Koo BK, Dietmann S, et al. Human primary liver cancer-derived organoid cultures for disease modeling and drug screening. Nat Med. 2017; 23:1424–35. 10.1038/nm.4438 29131160 PMC5722201

[24] Yaeger R, Chatila WK, Lipsyc MD, Hechtman JF, Cercek A, Sanchez-Vega F, Jayakumaran G, Middha S, Zehir A, Donoghue MTA, You D, Viale A, Kemeny N, et al. Clinical Sequencing Defines the Genomic Landscape of Metastatic Colorectal Cancer. Cancer Cell. 2018; 33:125–36.e3. 10.1016/j.ccell.2017.12.004 29316426 PMC5765991

[25] Bolhaqueiro ACF, Ponsioen B, Bakker B, Klaasen SJ, Kucukkose E, van Jaarsveld RH, Vivié J, Verlaan-Klink I, Hami N, Spierings DCJ, Sasaki N, Dutta D, Boj SF, et al. Ongoing chromosomal instability and karyotype evolution in human colorectal cancer organoids. Nat Genet. 2019; 51:824–34. 10.1038/s41588-019-0399-6 31036964

[26] Sansregret L, Vanhaesebroeck B, Swanton C. Determinants and clinical implications of chromosomal instability in cancer. Nat Rev Clin Oncol. 2018; 15:139–50. 10.1038/nrclinonc.2017.198 29297505

[27] Hause RJ, Pritchard CC, Shendure J, Salipante SJ. Classification and characterization of microsatellite instability across 18 cancer types. Nat Med. 2016; 22:1342–50. 10.1038/nm.4191 27694933

[28] Boland CR, Goel A. Microsatellite instability in colorectal cancer. Gastroenterology. 2010; 138:2073–87.e3. 10.1053/j.gastro.2009.12.064 20420947 PMC3037515

[29] Fakih MG, Kopetz S, Kuboki Y, Kim TW, Munster PN, Krauss JC, Falchook GS, Han SW, Heinemann V, Muro K, Strickler JH, Hong DS, Denlinger CS, et al. Sotorasib for previously treated colorectal cancers with KRAS^G12C^ mutation (CodeBreaK100): a prespecified analysis of a single-arm, phase 2 trial. Lancet Oncol. 2022; 23:115–24. 10.1016/S1470-2045(21)00605-7 34919824

[30] Jackstadt R, van Hooff SR, Leach JD, Cortes-Lavaud X, Lohuis JO, Ridgway RA, Wouters VM, Roper J, Kendall TJ, Roxburgh CS, Horgan PG, Nixon C, Nourse C, et al. Epithelial NOTCH Signaling Rewires the Tumor Microenvironment of Colorectal Cancer to Drive Poor-Prognosis Subtypes and Metastasis. Cancer Cell. 2019; 36:319–36.e7. 10.1016/j.ccell.2019.08.003 31526760 PMC6853173

[31] Marquart J, Chen EY, Prasad V. Estimation of the Percentage of US Patients With Cancer Who Benefit From Genome-Driven Oncology. JAMA Oncol. 2018; 4:1093–8. 10.1001/jamaoncol.2018.1660 29710180 PMC6143048

[32] Vlachogiannis G, Hedayat S, Vatsiou A, Jamin Y, Fernández-Mateos J, Khan K, Lampis A, Eason K, Huntingford I, Burke R, Rata M, Koh DM, Tunariu N, et al. Patient-derived organoids model treatment response of metastatic gastrointestinal cancers. Science. 2018; 359:920–6. 10.1126/science.aao2774 29472484 PMC6112415

[33] Sefrioui D, Beaussire L, Gillibert A, Blanchard F, Toure E, Bazille C, Perdrix A, Ziegler F, Gangloff A, Hassine M, Elie C, Bignon AL, Parzy A, et al. CEA, CA19-9, circulating DNA and circulating tumour cell kinetics in patients treated for metastatic colorectal cancer (mCRC). Br J Cancer. 2021; 125:725–33. 10.1038/s41416-021-01431-9 34112948 PMC8405627

[34] Drewinko B, Yand LY. Restriction of CEA synthesis to the stationary phase of growth of cultured human colon carcinoma cells. Exp Cell Res. 1976; 101:414–6. 10.1016/0014-4827(76)90393-1 964319

[35] Brattain MG, Fine WD, Khaled FM, Thompson J, Brattain DE. Heterogeneity of malignant cells from a human colonic carcinoma. Cancer Res. 1981; 41:1751–6. 7214343

[36] Meller B, Rave-Fränck M, Breunig C, Schirmer M, Baehre M, Nadrowitz R, Liersch T, Meller J. Novel Carcinoembryonic-Antigen-(CEA)-Specific Pretargeting System to Assess Tumor Cell Viability after Irradiation of Colorectal Cancer Cells. Strahlenther Onkol. 2011; 187:120–6. 10.1007/s00066-010-2191-5 21271227

[37] Golovko D, Kedrin D, Yilmaz ÖH, Roper J. Colorectal cancer models for novel drug discovery. Expert Opin Drug Discov. 2015; 10:1217–29. 10.1517/17460441.2015.1079618 26295972 PMC4872297

[38] Drewinko B, Yang LY, Barlogie B, Romsdahl M, Meistrich M, Malahy MA, Giovanella B. Further biologic characteristics of a human carcinoembryonic antigen-producing colon carcinoma cell line. J Natl Cancer Inst. 1978; 61:75–83. 10.1093/jnci/61.1.75 276641

[39] Rahaman MH, Lam F, Zhong L, Teo T, Adams J, Yu M, Milne RW, Pepper C, Lokman NA, Ricciardelli C, Oehler MK, Wang S. Targeting CDK9 for treatment of colorectal cancer. Mol Oncol. 2019; 13:2178–93. 10.1002/1878-0261.12559 31398271 PMC6763784

[40] Yu J, Liu D, Sun X, Yang K, Yao J, Cheng C, Wang C, Zheng J. CDX2 inhibits the proliferation and tumor formation of colon cancer cells by suppressing Wnt/β-catenin signaling via transactivation of GSK-3β and Axin2 expression. Cell Death Dis. 2019; 10:26. 10.1038/s41419-018-1263-9 30631044 PMC6328578

[41] Hua Q, Sun Z, Liu Y, Shen X, Zhao W, Zhu X, Xu P. KLK8 promotes the proliferation and metastasis of colorectal cancer via the activation of EMT associated with PAR1. Cell Death Dis. 2021; 12:860. 10.1038/s41419-021-04149-x 34552064 PMC8458432

[42] von Kleist S, Chany E, Burtin P, King M, Fogh J. Immunohistology of the antigenic pattern of a continuous cell line from a human colon tumor. J Natl Cancer Inst. 1975; 55:555–60. 10.1093/jnci/55.3.555 1159834

[43] Ahmed D, Eide PW, Eilertsen IA, Danielsen SA, Eknæs M, Hektoen M, Lind GE, Lothe RA. Epigenetic and genetic features of 24 colon cancer cell lines. Oncogenesis. 2013; 2:e71. 10.1038/oncsis.2013.35 24042735 PMC3816225

[44] Fantini J, Rognoni JB, Culouscou JM, Pommier G, Marvaldi J, Tirard A. Induction of polarized apical expression and vectorial release of carcinoembryonic antigen (CEA) during the process of differentiation of HT29-D4 cells. J Cell Physiol. 1989; 141:126–34. 10.1002/jcp.1041410119 2674159

[45] Ghadimi BM, Sackett DL, Difilippantonio MJ, Schröck E, Neumann T, Jauho A, Auer G, Ried T. Centrosome amplification and instability occurs exclusively in aneuploid, but not in diploid colorectal cancer cell lines, and correlates with numerical chromosomal aberrations. Genes Chromosomes Cancer. 2000; 27:183–90. 10612807 PMC4721570

[46] Drewinko B, Romsdahl MM, Yang LY, Ahearn MJ, Trujillo JM. Establishment of a human carcinoembryonic antigen-producing colon adenocarcinoma cell line. Cancer Res. 1976; 36:467–75. 1260746

[47] Rousset M, Chevalier G, Rousset JP, Dussaulx E, Zweibaum A. Presence and cell growth-related variations of glycogen in human colorectal adenocarcinoma cell lines in culture. Cancer Res. 1979; 39:531–4. 761227

[48] Verhoeckx K, Cotter P, López-Expósito I, Kleiveland C, Lea T, Mackie A, Requena T, Swiatecka D, Wichers H, editors. The Impact of Food Bioactives on Health: in vitro and ex vivo models. Cham (CH): Springer; 2015. 10.1007/978-3-319-16104-4 29787039

[49] Lelbovitz A, Wright WC, Pathak S, Siciliano MJ, Daniels WP. Detection and analysis of a glucose 6-phosphate dehydrogenase phenotype B cell line contamination. J Natl Cancer Inst. 1979; 63:635–45. 10.1093/jnci/63.3.635 288927

[50] Leibovitz A, Stinson JC, McCombs WB 3rd, McCoy CE, Mazur KC, Mabry ND. Classification of human colorectal adenocarcinoma cell lines. Cancer Res. 1976; 36:4562–9. 1000501

[51] Murakami H, Masui H. Hormonal control of human colon carcinoma cell growth in serum-free medium. Proc Natl Acad Sci U S A. 1980; 77:3464–8. 10.1073/pnas.77.6.3464 6932031 PMC349637

[52] Dharmsathaphorn K, McRoberts JA, Mandel KG, Tisdale LD, Masui H. A human colonic tumor cell line that maintains vectorial electrolyte transport. Am J Physiol. 1984; 246:G204–8. 10.1152/ajpgi.1984.246.2.G204 6141741

[53] Mouradov D, Sloggett C, Jorissen RN, Love CG, Li S, Burgess AW, Arango D, Strausberg RL, Buchanan D, Wormald S, O'Connor L, Wilding JL, Bicknell D, et al. Colorectal cancer cell lines are representative models of the main molecular subtypes of primary cancer. Cancer Res. 2014; 74:3238–47. 10.1158/0008-5472.CAN-14-0013 24755471

[54] Boyd D, Florent G, Murano G, Brattain M. Modulation of the urokinase receptor in human colon cell lines by N,N-dimethylformamide. Biochim Biophys Acta. 1988; 970:96–100. 10.1016/0167-4889(88)90227-3 2835992

[55] Sparks AB, Morin PJ, Vogelstein B, Kinzler KW. Mutational analysis of the APC/beta-catenin/Tcf pathway in colorectal cancer. Cancer Res. 1998; 58:1130–4. 9515795

[56] Walther A, Houlston R, Tomlinson I. Association between chromosomal instability and prognosis in colorectal cancer: a meta-analysis. Gut. 2008; 57:941–50. 10.1136/gut.2007.135004 18364437

[57] Hong DS, Fakih MG, Strickler JH, Desai J, Durm GA, Shapiro GI, Falchook GS, Price TJ, Sacher A, Denlinger CS, Bang YJ, Dy GK, Krauss JC, et al. KRAS^G12C^ Inhibition with Sotorasib in Advanced Solid Tumors. N Engl J Med. 2020; 383:1207–17. 10.1056/NEJMoa1917239 32955176 PMC7571518

[58] Sotorasib's Benefits in Colorectal Cancer Modest. Cancer Discov. 2022; 12:OF1. 10.1158/2159-8290.CD-NB2022-0001 34987029

[59] Awad MM, Liu S, Rybkin II, Arbour KC, Dilly J, Zhu VW, Johnson ML, Heist RS, Patil T, Riely GJ, Jacobson JO, Yang X, Persky NS, et al. Acquired Resistance to KRAS^G12C^ Inhibition in Cancer. N Engl J Med. 2021; 384:2382–93. 10.1056/NEJMoa2105281 34161704 PMC8864540

[60] Zhao Y, Murciano-Goroff YR, Xue JY, Ang A, Lucas J, Mai TT, Da Cruz Paula AF, Saiki AY, Mohn D, Achanta P, Sisk AE, Arora KS, Roy RS, et al. Diverse alterations associated with resistance to KRAS(G12C) inhibition. Nature. 2021; 599:679–83. 10.1038/s41586-021-04065-2 34759319 PMC8887821

[61] Dienstmann R, Tabernero J. Cancer: A precision approach to tumour treatment. Nature. 2017; 548:40–1. 10.1038/nature23101 28723897

[62] Bürtin F, Mullins CS, Linnebacher M. Mouse models of colorectal cancer: Past, present and future perspectives. World J Gastroenterol. 2020; 26:1394–426. 10.3748/wjg.v26.i13.1394 32308343 PMC7152519

[63] Tetteh PW, Kretzschmar K, Begthel H, van den Born M, Korving J, Morsink F, Farin H, van Es JH, Offerhaus GJ, Clevers H. Generation of an inducible colon-specific Cre enzyme mouse line for colon cancer research. Proc Natl Acad Sci U S A. 2016; 113:11859–64. 10.1073/pnas.1614057113 27708166 PMC5081651

[64] Chuprin J, Buettner H, Seedhom MO, Greiner DL, Keck JG, Ishikawa F, Shultz LD, Brehm MA. Humanized mouse models for immuno-oncology research. Nat Rev Clin Oncol. 2023; 20:192–206. 10.1038/s41571-022-00721-2 36635480 PMC10593256

[65] Chang A, Liu L, Ashby JM, Wu D, Chen Y, O'Neill SS, Huang S, Wang J, Wang G, Cheng D, Tan X, Petty WJ, Pasche BC, et al. Recruitment of KMT2C/MLL3 to DNA Damage Sites Mediates DNA Damage Responses and Regulates PARP Inhibitor Sensitivity in Cancer. Cancer Res. 2021; 81:3358–73. 10.1158/0008-5472.CAN-21-0688 33853832 PMC8260460

[66] Na F, Pan X, Chen J, Chen X, Wang M, Chi P, You L, Zhang L, Zhong A, Zhao L, Dai S, Zhang M, Wang Y, et al. KMT2C deficiency promotes small cell lung cancer metastasis through DNMT3A-mediated epigenetic reprogramming. Nat Cancer. 2022; 3:753–67. 10.1038/s43018-022-00361-6 35449309 PMC9969417

[67] Katsiampoura A, Raghav K, Jiang ZQ, Menter DG, Varkaris A, Morelli MP, Manuel S, Wu J, Sorokin AV, Rizi BS, Bristow C, Tian F, Airhart S, et al. Modeling of Patient-Derived Xenografts in Colorectal Cancer. Mol Cancer Ther. 2017; 16:1435–42. 10.1158/1535-7163.MCT-16-0721 28468778 PMC5562363

[68] Cheng F, Wan X, Wang B, Li Y, Peng P, Xu S, Han C, Mao F, Guo D. Establishment and characteristics of GWH04, a new primary human glioblastoma cell line. Int J Oncol. 2022; 61:139. 10.3892/ijo.2022.5429 36169178 PMC9529431

[69] Shaffer LG, McGowan-Jordan J, Schmid M. ISCN 2013: An International System for Human Cytogenetic Nomenclature (2013). Published in Collaboration with 'Cytogenetic and Genome Research'. 2012.

[70] Liao Z, Chen L, Zhang X, Zhang H, Tan X, Dong K, Lu X, Zhu H, Liu Q, Zhang Z, Ding Z, Dong W, Zhu P, et al. PTPRε Acts as a Metastatic Promoter in Hepatocellular Carcinoma by Facilitating Recruitment of SMAD3 to TGF-β Receptor 1. Hepatology. 2020; 72:997–1012. 10.1002/hep.31104 31903610

[71] Zhang B, Halder SK, Zhang S, Datta PK. Targeting transforming growth factor-beta signaling in liver metastasis of colon cancer. Cancer Lett. 2009; 277:114–20. 10.1016/j.canlet.2008.11.035 19147275 PMC2776056

