# Supplementary material for: A pair of primary colorectal cancer-derived and corresponding synchronous liver metastasis-derived organoid cell lines
Source: Aging (Albany NY). 2024 Feb 24;16(5):4396–422. doi: 10.18632/aging.205595 (PMC10968669; doi:10.18632/aging.205595)
Supplement: Supplementary Figure 1 [file aging-16-205595-s001.pdf]

SUPPLEMENTARY FIGURE

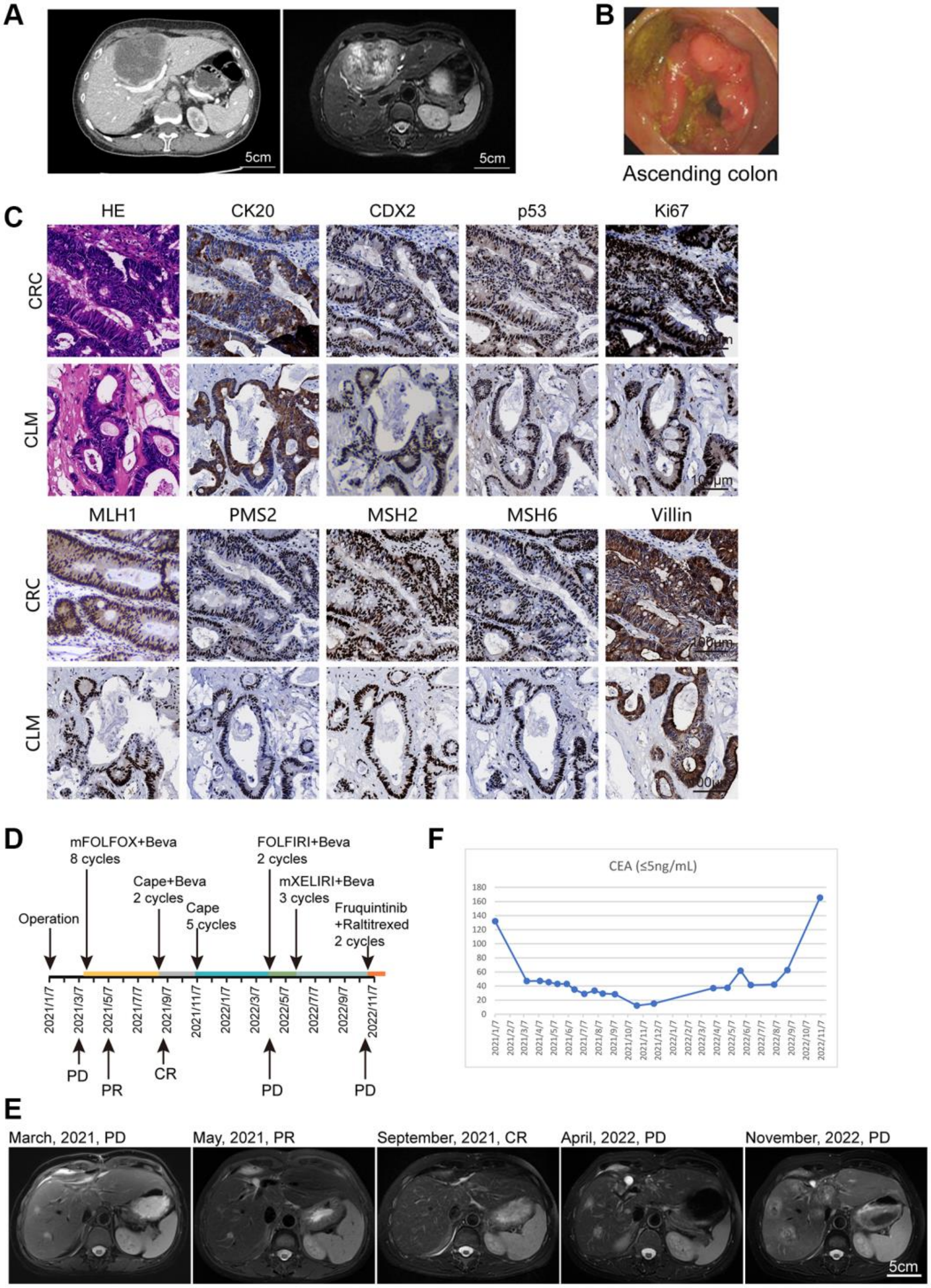

Supplementary Figure 1. Clinical features of the patient from whom CWH22 and CLM22 organoids were derived. (**A**) CT (venous phase) and MRI (T2-weighted) scans of lesions on the left lobe of the liver (scale bars, 5 cm). (**B**) Image of the space-occupying lesion in the ascending colon under colonoscopy. (**C**) H&E and IHC staining used for histological diagnosis. Scale bars, 100 μm. (**D**) Timeline of treatments administered to the patient and treatment response records (PD: progressive disease; PR: partial response; CR: complete response). (**E**) Representative MR images of the liver at different treatment response time points. (**F**) Serum CEA levels during the treatment period from January 2021 to November 2022, reflecting the treatment response.
