# Supplementary material for: A pair of primary colorectal cancer-derived and corresponding synchronous liver metastasis-derived organoid cell lines
Source: Aging (Albany NY). 2024 Feb 24;16(5):4396–422. doi: 10.18632/aging.205595 (PMC10968669; doi:10.18632/aging.205595)
Supplement: Supplementary Table 1 [file aging-16-205595-s002.pdf]

SUPPLEMENTARY TABLE

Supplementary Table 1. Basic information of KRAS unmutated and G12D mutated organoids.

Org	Diagnosis	Stage	MSI/MSS	KRAS	APC	SMAD4	Tp53	PIK3CA
CRC1-Org	Sigmoid colon cancer	IIA	MSI	Missense	Nonsense	Wild-type	Missense	Missense
CRC16-Org	Ascending colon carcinoma, Liver Metaseases, Peritoneum metastases	IVB	MSS	Missense	Wild-type	Wild-type	Missense	Missense
CRC27-Org	Sigmoid colon cancer, Liver metastases	IVA	MSS	Wild-type	Missense	Wild-type	Missense	Wild-type
